# Insights Gained by High-throughput Chromosome Conformation Capture (Hi-C) into the Viral Modulation of Methane Production in Anaerobic Digestion

**DOI:** 10.1021/acs.est.6c03952

**Published:** 2026-07-09

**Authors:** Junya Zhang, Tiedong Lu, Yunwei Cui, Qihe Tang, Yuansong Wei, Hans Hermann Richnow

**Affiliations:** † State Key Laboratory of Regional Environment and Sustainability, Research Center for Eco-Environmental Sciences, 26442 Chinese Academy of Sciences, Beijing 100085, China; ‡ Agricultural Resource and Environment Research Institute, 125388 Guangxi Academy of Agricultural Sciences/Guangxi Key Laboratory of Arable Land Conservation, Nanning 530007, China; § Atmospheric Chemistry Department (ACD), Leibniz Institute of Tropospheric Research (TROPOS), Permoserstraße 15, Leipzig 04318, Germany; ∥ College of Life Science and Technology, Guangxi University, Nanning 530005, China; ⊥ University of Chinese Academy of Sciences, Beijing 100049, China

**Keywords:** methane metabolism, viral community, auxiliary metabolic genes, anaerobic digestion, high-throughput chromosome conformation capture sequencing

## Abstract

Methanogenesis is a critical driver of global carbon cycling and bioenergy recovery, yet how bacteriophages modulate methane production remains poorly understood. Here, we integrated high-throughput chromosome conformation capture (Hi-C) with multi-omics to map in situ phage–host interactions through an over 440 day anaerobic digestion experiment. We captured 6100 active physical linkages, revealing that 16.3 ± 2.1% of interactions involved auxiliary metabolic genes (AMGs). Notably, we observed a dynamic community-level compositional shift in viral life strategies driven by operational stress. Under mesophilic conditions, lysogenic piggyback-the-winner dynamics prevailed, with AMGs enhancing host competitiveness. Conversely, thermophilic conditions with high total solids stress triggered lytic kill-the-winner strategies. During this phase, AMGs supported rapid phage replication targeting overproliferating bacteria to restore the disrupted balance between acidogenesis and methanogenesis. Furthermore, we identified 529 DNA viral operational taxonomic units (vOTUs) directly infecting methanogens, alongside broad-host-range phages spanning bacterial and archaeal domains. Importantly, we detected four RNA vOTUs exclusively under thermophilic conditions, providing the first omics-based evidence of RNA phages actively infecting methanogenic archaea. These findings highlight phages as important modulators of methane production, offering a foundational framework for developing targeted phage-engineering strategies to optimize bioenergy systems and mitigate methane emissions.

## Graphical abstract

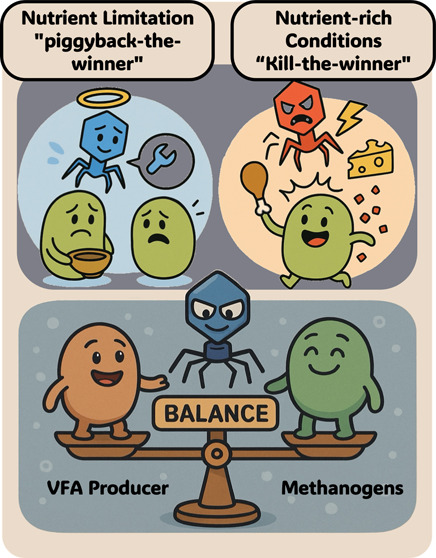

## Introduction

Methane (CH_4_) is a critical driver of global climate change, with a warming potential over 25 times greater than carbon dioxide (CO_2_) over a 100 year time scale, contributing approximately 20% to global radiative forcing.^1,2^ Biological methanogenesis, primarily mediated by archaea in anoxic environments, accounts for ∼70% of global CH_4_ emissions, releasing an estimated 300–450 Tg annually.^3^ This process, encompassing hydrogenotrophic, acetoclastic, and methylotrophic pathways, converts simple substrates such as CO_2_, H_2_, and acetate into CH_4_, playing a pivotal role in carbon cycling across ecosystems like wetlands, ruminant guts, and engineered bioreactors.^4^ Methanogenic archaea often operate in syntrophy with bacteria, maintaining low partial pressures of fermentation byproducts to sustain energetically favorable conditions for organic matter degradation.^5^ This breakdown of organic matter to methane accounts for a substantial proportion of total carbon cycling on Earth, equivalent to 2% of all CO_2_ fixed annually.^6^ As Earth warms at an unprecedented rate, understanding the microbial interactions that regulate methanogenesis is essential for predicting CH_4_ fluxes and developing strategies to mitigate greenhouse gas emissions while optimizing bioenergy production.

Viruses, particularly bacteriophages (phages), are ubiquitous ecological agents, with an estimated global abundance of 10^31^ particles and driving 20–40% of microbial mortality daily through ∼10^23^ infections per second.^7,8^ Phages shape microbial communities via predation, horizontal gene transfer, and metabolic modulation, employing strategies such as “kill-the-winner,” where dominant populations are lysed, or “piggyback-the-winner,” where lysogenic phages integrate into thriving hosts.^8–10^ In methanogenic environments, phages encode auxiliary metabolic genes (AMGs) that can enhance host resilience to environmental stressors, such as nutrient limitation or pH shifts, potentially amplifying methanogenic activity. For instance, AMGs linked to carbon and sulfur metabolism have been identified in methane-rich habitats, yet their specific roles in CH_4_ metabolism remain elusive.^2^ Current knowledge of phage–methanogen interactions is limited by reliance on indirect methods, such as CRISPR-Cas spacer matching or sequence-based predictions,^11–13^ which fail to capture active infections at the time of sampling. This gap hinders our ability to elucidate the molecular and ecological mechanisms by which phages influence CH_4_ cycling, constraining predictive models and emission control strategies. To circumvent these challenges, high-throughput chromosome conformation capture (Hi-C) sequencing has recently emerged as a revolutionary approach in viral ecology.^14,15^ By physically cross-linking viral and host DNA in situ within intact cells prior to lysis, Hi-C can directly map active, ongoing virus–host infection networks and resolve broad-host-range viral traits within complex microbiomes. Leveraging this advanced technology is, therefore, essential to transition from historical or theoretical sequence matching to the characterization of real-time, active viral modulation in methanogenic environments.

Anaerobic digestion (AD) systems, pivotal for waste-to-energy conversion, offer a controlled platform to study phage-mediated methanogenesis. Processing organic substrates like manure and food waste, AD reactors produce biogas with 45–75% CH_4_, driven by microbial consortia catalyzing hydrolysis, acidogenesis, acetogenesis, and methanogenesis.^16^ With ∼20,000 biogas plants in Europe alone and tens of thousands of AD facilities globally, these systems generated ∼1.3 million TJ of energy per year, reflecting a 13.2% annual growth since 2000.^17^ Full utilization of sustainable feedstocks could meet 20% of global gas demand, avoiding ∼1000 Mt of greenhouse gas emissions by 2040.^16^ While methanogenic archaea and their bacterial partners are well-studied, phage roles, potentially *via* AMG-mediated metabolic enhancement or selective lysis, remain largely unexplored. Unraveling these interactions could optimize CH_4_ yields and mitigate emissions, with profound implications for global carbon cycling.

To address the critical question of how phages regulate methanogenesis, we conducted an over 440 day semi-continuous AD experiment using animal manure (second to crop residuals considering the global production potential for biogas or biomethane^16^), systematically evaluating the effects of operational perturbations iron-based compound amendments, extended sludge retention time (SRT), temperature shifts from 37 to 55 °C, and increased organic loading rates) on CH_4_ metabolism. We integrated Hi-C sequencing with metagenomics and meta-transcriptomics to capture active phage–host interactions and characterize DNA and RNA viral communities. Hi-C, which chemically cross-links viral and host DNA during infection,^15,18^ enabled us to distinguish ongoing “piggyback-the-winner” dynamics together with the lifestyle prediction, where AMGs bolster methanogens, from “kill-the-winner” lysis events that regulate community structure. Metagenomics identified AMGs and functional genes linked to methanogenesis, while meta-transcriptomics validated their expression under varying conditions. Additionally, CRISPR-Cas profiling reconstructed historical phage–methanogen interactions, providing a temporal context for infection dynamics. By linking these molecular insights to CH_4_ fluxes, our study establishes a mechanistic framework for phage-mediated methanogenesis, offering novel perspectives on microbial contributions to global CH_4_ cycling and informing strategies for bioenergy optimization and greenhouse gas mitigation.

## Materials and Methods

### Experimental Setup

Three identical continuous stirred-tank reactors (10 L total volume, 8 L working volume) were operated semicontinuously for over 440 days to evaluate the effects of iron amendments, SRT, temperature, and total solids (%, TS) on phage-mediated methanogenesis during AD of swine manure (Figure 1a). Reactors were inoculated with digested swine manure sludge (inoculum-to-feed ratio 1:3 *w*/*w*) at startup. Swine manure (TS ≈ 25–30%) was collected weekly from the same farm, diluted to target TS, and stored at 4 °C before use. The experiment comprised six sequential stages, each lasting ≥3 SRTs.

- Stage I (days 0–45): Startup; TS 10%, SRT 15 d, 37 °C, feeding every 3 d (1.6 L fresh manure in, 1.6 L digestate out), agitation 80 rpm (30 min on/15 min off).
- Stage II (days 46–90): Reactors differentiated without other changes: CK (control), R1 (75 mmol L^–1^ Fe_2_O_3_ nanoparticles, Aladdin F108317, <50 nm), R2 (5 mmol L^–1^ FeCl_3_·6H_2_O, Aladdin I112065). Iron compounds were premixed with daily feed to maintain constant concentrations (concentrations previously optimized for ARG attenuation and methane enhancement^19,20^ ).
- Stage III (days 91–171): SRT extended to 24 d without other changes (all reactors).
- Stage IV (days 172–244): Temperature increased to 55 °C without other changes (thermophilic, all reactors).
- Stage V (days 245–340): TS increased to 15% without other changes (all reactors).
- Stage VI (days 342–440): TS further increased to 20% without other changes (all reactors).

**Figure 1:**
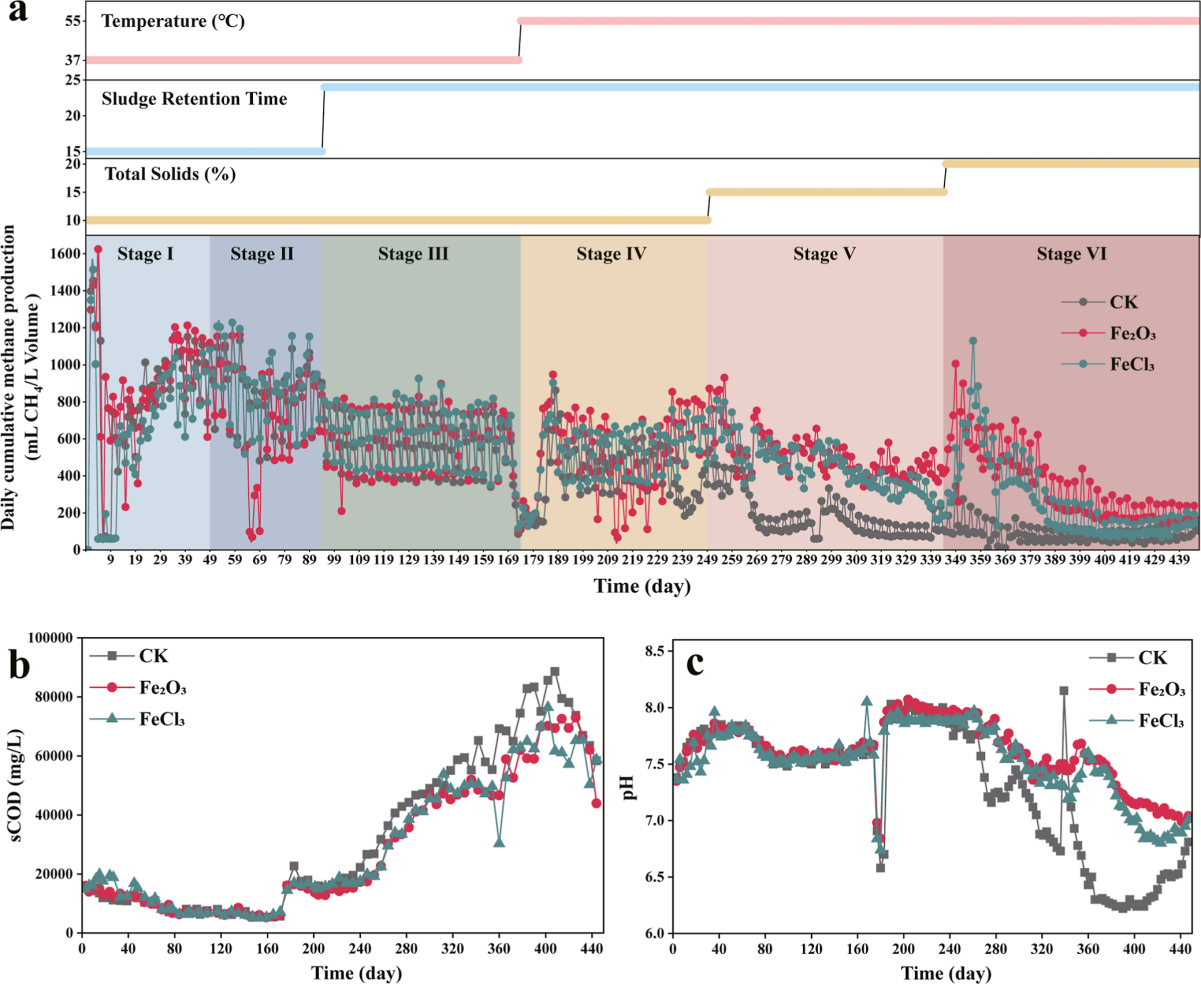
Operational parameters and performance of the anaerobic digestion experiment. (a) Experimental timeline showing temperature, sludge retention time (SRT, days), and total solids (TS, %) across six stages, together with daily methane production in the three reactors (CK, control; R1, Fe_2_O_3_-amended; R2, FeCl_3_-amended). (b) Soluble chemical oxygen demand (sCOD) dynamics. (c) pH dynamics.

The pH and SCOD were monitored biweekly by using standard methods. Daily methane production was automatically and continuously measured using a microgas flow meter (BPC Instruments, Sweden) after the biogas was passed through a gas-washing bottle containing a 3 M NaOH solution to scrub the CO_2_. Samples were collected at least once per SRT for short-read metagenomics and meta-transcriptome analyses, with composite stage samples processed for Hi-C and long-read nanopore sequencing.

### DNA and RNA Extraction

The experimental time comprises a total of 20 SRTs (6 SRTs of 15 days and 14 SRTs of 24 days) for each reactor, yielding 60 samples plus the day 0 (D0) sample for DNA extraction and short-read metagenomic sequencing. Starting from Stage II after startup, meta-transcriptome samples (15 in total) were collected at the end of each stage where steady-state conditions were achieved better, while the five composite stage samples (mixing the samples from CK, R1, and R2 in the same stage) were used for the Hi-C and long-read nanopore sequencing. This pooling strategy enabled long-read sequencing as a foundational reference to improve the completeness of metagenome-assembled genomes (MAGs) and viral operational taxonomic units (vOTUs) and allowed Hi-C to capture a broader, more comprehensive network of active phage–host interactions representative of overarching stage parameters. DNA extraction was done using the FastDNA Spin kit for soil (MP Bio, USA), while RNA was extracted through the Soil RNA Kit200 (Omega BioTek, GA, USA), followed by rRNA removal with the Ribo-Zero rRNA Removal Kit (Epicenter, Madison, WI, USA). Extracted DNA and RNA were detected and quantified by electrophoresis on a 1% agarose gel and a NanoDrop 2000 (Thermo Scientific, USA), respectively.

### Short- and Long-Read Metagenomics along with Meta-Transcriptome Sequencing

These DNA and RNA samples were sent to Majorbio BioPharm Technology Co., Ltd. (Shanghai, China) for library construction (350 bp) and pair-end sequencing (150 bp). The meta-transcriptome library construction (350 bp) was prepared using TruSeqTM RNA Sample Prep Kit. Both metagenomics and meta-transcriptomic sequencing were conducted through the HiSeq 4000 platform (Illumina, USA). Long-read libraries were prepared using Oxford Nanopore Technologies (ONT) Ligation library preparation kit (SQK-LSK109, EXP-NBD104, and EXP-NBD114) following manufacturer’s instructions and sequenced with the ONT PromethION sequencer using FLO-PRO002 flow cells. Approximately 1.01 Tb, 451 Gb, and 63.5 Gb of raw data were generated for the short-read metagenomics, meta-transcriptome and long-read nanopore sequencing, respectively.

### Profiling of Methane Metabolism Genes

Short-read metagenomics and meta-transcriptomics were both trimmed and quality-controlled through a metaWRAP-Read_qc module.^21^ Methane metabolism (MM) genes-like reads were determined by Diamond (v2.0.14, −query-cover 75, −id 90, −*e*-value 1 × 10^–5^) against MCycDB.^22^ To reduce the bias caused by the sequencing depth and biomass, the expression of MM genes at the RNA level was normalized by reference length and sequencing depth as RPKM (reads per kilobase per million reads), while the relative abundance of MM genes at the DNA level was further normalized by RPKM of 16S rRNA.

The clean reads were assembled using MEGAHIT (v1.1.3, -mini-contig-len 1000) individually. MAGs based on short- and long-read metagenomics were collected as follows: (1) by binning using metaBAT2, MaxBin2, and CONCOCT; (2) by refining through a Bin_refinement module in MetaWRAP (v1.3.0) with completeness >80% and contamination <5%; and (3) by combining and dereplicating through dRep.^23^ To improve MAG quality, we identified and removed contaminating contigs from each MAG using MAGpurify v2.1.2 with the default modules and parameters.^24^ The average completeness and contamination of the final collected MAGs are 91.6% and 1.6%, respectively, which indicated the high quality of these MAGs according to Minimum Information for a Metagenome-Assembled Genome (MIMAG) standards.^25^ MAGs were taxonomically classified by GTDB-Tk (v1.5.0, R214).^26^ The MAGs were annotated through distilled and refined annotation of metabolism (DRAM) against the Kyoto Encyclopedia of Genes and Genomes (KEGG) and PFAM databases by the default parameters. The results were further used for recovering microbial MM genes based on their KEGG and PFAM annotations against the KEGG MM pathway modules.^2,27^

### Viral Contigs Identification, Clustering, and Taxonomic Assignment

Viral contigs were identified, clustered, and taxonomic-assigned, as described in our previous study.^28^ Briefly, DNA contigs >5.0 kb were collected, dereplicated, and then piped through VirSorter2, VirFinder, and geNomad,^29–31^ which were then merged and dereplicated with CD-HIT (v4.7). The valid 13,124 viral contigs were subjected to species-level clustering to create vOTUs using the ClusterGenomes scripts with 95% average nucleotide identity (ANI) and 85% alignment fraction (AF),^32^ resulting in 6,210 vOTUs. The composite stage ONT long-reads were used to improve the quality of these vOTUs. ONT long-reads were individually assembled with metaFlye (v2.9-b1768.32) into contigs. Long-read viral contigs were identified, combined, and dereplicated as previously described. Quickmerge was employed to improve the quality of vOTUs with the 6,210 vOTUs as the reference.

Taxonomic assignment of these vOTUs was carried out using five methods: (1) vConTACT2;^33^ (2) majority-rules approach;^34,35^ (3) blastn against Integrated Microbial Genome/Virus (IMG/VR, v3.0) and RefSeq virus database (release 203); (4) CAT;^36^ and (5) geNomad.^31^ The viral lifestyle was predicted by integrating the results of CheckV, VIBRANT, and PhaTYP. CheckV identified temperate viruses by detecting proviral integration sites or integrase genes.^37^ VIBRANT (v1.2.1) is based on the hybrid machine learning and protein similarity approach,^38^ and PhaTYP employed machine learning to predict lifestyle based on characteristic protein composition and association patterns.^39^ A vOTU was classified as temperate if it met at least one of the following criteria: identified as a provirus by CheckV, labeled “lysogenic” by VIBRANT, or assigned a temperate score ≥0.8 by PhaTYP. vOTUs that did not fulfill any of these criteria were considered potentially lytic/virulent.^40^ The predicted viral ORFs were also compared to the IMG/VR v3 and RefSeq viral protein database using DIAMOND (-id 30, -query-cover 50, -evalue 1 × 10^–5^) to verify the novelty of the collected vOTUs.

Besides, we also identified the RNA viruses based on the meta-transcriptomics through the RdRp homology and geNomade (gene marker and machine learning),^31,41^ where 517 RNA vOTUs were finally identified (Supporting Information Text S1). Although most of them belonged to pathogenic viruses infecting eukaryotes, 89 RNA vOTUs belonged to the phages.

### Auxiliary Metabolism Genes Identification

As no standardized pipeline currently exists for AMG identification, we compared two widely used tools: VIBRANT^38^ and DRAM-v.^27^ VIBRANT (default settings) was applied to all 6,210 DNA vOTUs and 517 RNA vOTUs. Predicted AMGs were manually inspected to confirm the presence of viral genes both upstream and downstream of the candidate. DRAM-v (default settings) was run on the 5,418 DNA vOTUs that passed the VirSorter2 classification (required input for DRAM-v). DRAM-v provides a built-in auxiliary score that evaluates how confidently a gene is viral based on the neighboring viral genes. We retained only putative AMGs with an auxiliary score of <4 (indicating high viral confidence) and that lacked any of the following flags: viral flag (F), transposon flag (T), viral-like peptidase flag (P), or attachment flag (A). Candidates without a gene ID or a functional description were discarded. All vOTUs were additionally annotated with DRAM using the KEGG and PFAM databases (default parameters). These annotations were subsequently used to identify MM genes based on the KEGG MM pathway (map00680). We noticed the call for caution in the biological interpretation of viral AMGs,^42^ and we carefully checked the AMGs here (see Supporting Information for more information).

Because most AMGs are horizontally acquired from hosts and could therefore be confounded by contaminating host reads, we used vOTU abundance as a proxy for the abundance of their encoded AMGs.^43^ Viral contig abundance in each sample was calculated as RPKM using CoverM. To estimate AMG expression while minimizing contribution from host-derived transcripts, we quantified short-read mapping to AMGs at 100% nucleotide identity and 100% query coverage, again reporting expression as RPKM.

### Hi-C Construction of Ongoing Virus–Host Infections

Hi-C libraries for Stages II–VI were prepared from composite stage samples using the ProxiMeta Hi-C preparation kit (Phase Genomics, Seattle, WA, USA). DNA was digested in situ with Sau3AI and proximity-ligated; libraries were sequenced on an Illumina HiSeq 4000 (2 × 150 bp, ∼30 Gb per sample). Then, the phage–host interactions were established as follows: (1) To establish strict contact filtering criteria, Hi-C reads were quality-filtered as described for the metagenomes and aligned to a combined reference of all collected vOTUs and MAGs using BWA-MEM with the −5SP parameter, which is explicitly specified to reduce secondary and alternative mappings. Furthermore, secondary, supplementary, and unmapped alignments were discarded (samtools view –F 0 × 904), and the files were name-sorted to retain only unique, high-quality alignments. (2) As a normalization procedure, the NormCC module from the MetaCC pipeline was adopted to eliminate the systematic biases inherent in Hi-C contacts. (3) To establish robust linkage thresholds, an active virus–host linkage was called only when a minimum threshold of ≥5 normalized Hi-C links connected a vOTU and a MAG. (4) Finally, to mitigate false-positive ligations and ensure physical co-occurrence, these linkages were validated only if both the vOTU and the MAG were simultaneously present with a high genomic coverage (≥70% base coverage) in at least one sample of that operational stage. Viral copies per host cell (VPH) were calculated from normalized Hi-C link counts, vOTU abundance (V), and host abundance (H) as previously described.^15^

### Historical (In-Memory) Virus–Host Linkages

Historical linkages were inferred using three complementary approaches.^28^ (1) CRISPR-Cas spacers extracted from MAGs were searched against vOTUs with BLASTN-short (100% identity, 100% coverage).^15^ (2) The vOTU-encoded tRNAs were aligned to MAGs (100% identity, 100% coverage, no self-hits and duplicates). (3) Nucleotide homology was assessed by BLASTN (≥80% identity, alignment length between 1 kb and 50% of the microbial host contig, *E*-value ≤10^–5^).^44,45^ The abundance and expression of target sequences (spacers, genes, contigs, and MAGs) were quantified as RPKM using CoverM.^46^

### Statistical Analysis

All statistical analyses and visualizations were conducted with R (version 4.3.2). The differences in daily methane yields, gene abundances, and transcript expression levels across the different operational stages were evaluated using the nonparametric Kruskal–Wallis test, and a *P* value of less than 0.05 was considered statistically significant. Mantel tests and Procrustes analyses (implemented via the *vegan* package) were performed to evaluate the correlations between viral and bacterial community structure variations. Pearson correlation coefficients (*r*) were calculated to assess the linear relationships between the relative abundances of specific vOTUs and interacting MAGs.

## Results

### Methane Production in AD

We investigated the impact of operational parameters on methane production in AD through a 440 day semi-continuous experiment using swine manure as the substrate (Figure 1a). Three experimental groups were established: a control (CK) and two iron-supplemented groups (R1: Fe_2_O_3_; R2: FeCl_3_). We evaluated the daily methane yields under varying conditions: mesophilic (35 °C) and thermophilic (55 °C) temperatures, SRTs of 15 and 24 days, and TS contents of 15% and 20%. Under mesophilic conditions (Stages I–II), daily volumetric methane yields averaged 681.6 ± 218.3 mL CH_4_ L^–1^ across all groups, with no significant differences among CK, R1, and R2. Extending SRT from 15 to 24 days (Stage III) significantly reduced daily yields from 822.5 ± 236.0 to 603.3 ± 161.4 mL CH_4_ L^–1^ (Kruskal–Wallis test, *P* < 0.001), likely due to substrate limitation, which can also be seen through the decline of sCOD in the AD system (Figure 1b). Transitioning to thermophilic conditions (55 °C, Stage IV) further decreased yields to 276.0 ± 98.5 mL CH_4_ L^–1^ across groups. However, after microbial adaptation (10 days in R1/R2, ∼13 days in CK), daily yields recovered to levels comparable to late mesophilic Stage III, with no subsequent increase above mesophilic performance. This pattern aligns with the similar ultimate methane potential of swine manure at 37 and 55 °C in previous studies,^47,48^ and we think the faster kinetics at thermophilic conditions are offset by longer SRT (24 d) already sufficient for near-complete substrate conversion, plus elevated free ammonia inhibition at thermophilic temperatures. Iron amendments accelerated community adaptation to the thermal stress. Increasing TS to 15% (Stage V) and 20% (Stage VI) triggered progressive decline, with CK daily yields dropping to 194.8 ± 123.4 and 89.7 ± 53.0 mL CH_4_ L^–1^, respectively, accompanied by pH decline (Figure 1c). Late-stage decrease in sCOD despite persistently low methane production reflects reduced solubility and hydrolysis of particulate organics at 20% TS rather than improved substrate utilization. This is consistent with established high-solids AD paradigms, where severely restricted free water and increased mass transfer limitations at elevated TS levels profoundly impede enzymatic access and subsequent hydrolysis.^49^ Iron-supplemented reactors maintained significantly higher daily yields (496.1 ± 131.0 and 308.2 ± 200.4 mL CH_4_ L^–1^). These results indicate that iron compounds (Fe_2_O_3_ and FeCl_3_) mitigate the inhibitory effects of high organic loading at thermophilic conditions, likely by stabilizing microbial consortia or facilitating electron transfer.

In summary, extended SRT and the initial thermophilic shift reduced methane yields, but iron supplementation accelerated recovery from temperature stress and sustained higher yields under elevated organic loading (15–20% TS).

### MM Genes in AD

We assembled and refined 312 high-quality MAGs from AD samples with an average completeness of 91.6% and contamination of 1.6%. These high-quality MAGs accounted for approximately 60% of sequencing reads per sample, ensuring robust community representation. Annotation using DRAM identified 4,878 MM genes across all MAGs (average 15.6 per MAG, Table S1), encompassing complete KEGG pathway modules and 146 KOs (KEGG orthology) for MM (Figure S1). Every MAG harbored at least three MM genes, confirming the widespread involvement in methane cycling in the specific AD system. Of the 13 archaeal MAGs recovered, 10 were methanogens, carrying significantly more MM genes (mean 87.5) than nonmethanogenic MAGs (mean 13.2). Notably, MAG207 (*Methanosarcina mazei*) and MAG146 (*Methanosarcina thermophila*) encoded 126 and 116 MM genes, respectively. The methyl-coenzyme M reductase (Mcr) enzyme, central to methanogenesis, is a key target for probing CH_4_ fluxes,^6,50^ and the marker gene *mcrA* was detected in seven methanogenic MAGs, with the remaining three encoding other *mcr* operoncomponents, underscoring the central role of methanogenic archaea in driving methane production in AD.

Metagenomic analysis at the read level revealed that MM gene relative abundances were dominated by the serine cycle (39.0%), followed by the central methanogenic pathway (21.0%), RuMP cycle (16.0%), and acetoclastic methanogenesis (15.0%) (Figure 2a). The serine and RuMP cycles primarily support methane assimilation and carbon fixation, whereas the central methanogenic pathway and acetoclastic methanogenesis drive methane production, with acetoclastic methanogenesis predominating over hydrogenotrophic (2.0%) and methylotrophic (2.0%) pathways, reflecting acetate as a key substrate in manure-based AD. Key genes included *eno* and *glyA* in the serine cycle, *pfkA* and *fbaA* in the RuMP cycle, *ackA*, *pta*, and *acdA* in acetoclastic methanogenesis, and *rnfA* in the central methanogenic pathway (Figure 2b). Extending SRT from 15 to 24 days (Stage III) had minimal impact on MM gene composition, despite the observed reduction in daily methane yields. This observation further suggests that substrate limitation, as reflected by the lower sCOD, was the primary driver of the yield declines (Figure 1b). Transition to thermophilic conditions (55 °C, Stage IV) significantly reduced MM gene abundances (Kruskal–Wallis test, *P* < 0.001), particularly in the central methanogenic pathway (e.g., *mcrA* and *mtrA*) and acetoclastic methanogenesis (e.g., *ackA*, *pta*, and *acdA*). Increasing TS to 15% (Stage V) and 20% (Stage VI) elevated MM gene abundances across all modules (Kruskal–Wallis test, *P* < 0.001), indicating enhanced microbial activity under higher organic loading. Notably, iron compounds did not significantly alter MM gene relative abundances, suggesting their role in enhancing methane production (as observed in Stage IV–VI) likely stems from modulating the AD microenvironment (Figure 1c, e.g., pH stabilization or direct interspecies electron transfer) or post-transcriptional effects rather than genomic abundance.

**Figure 2:**
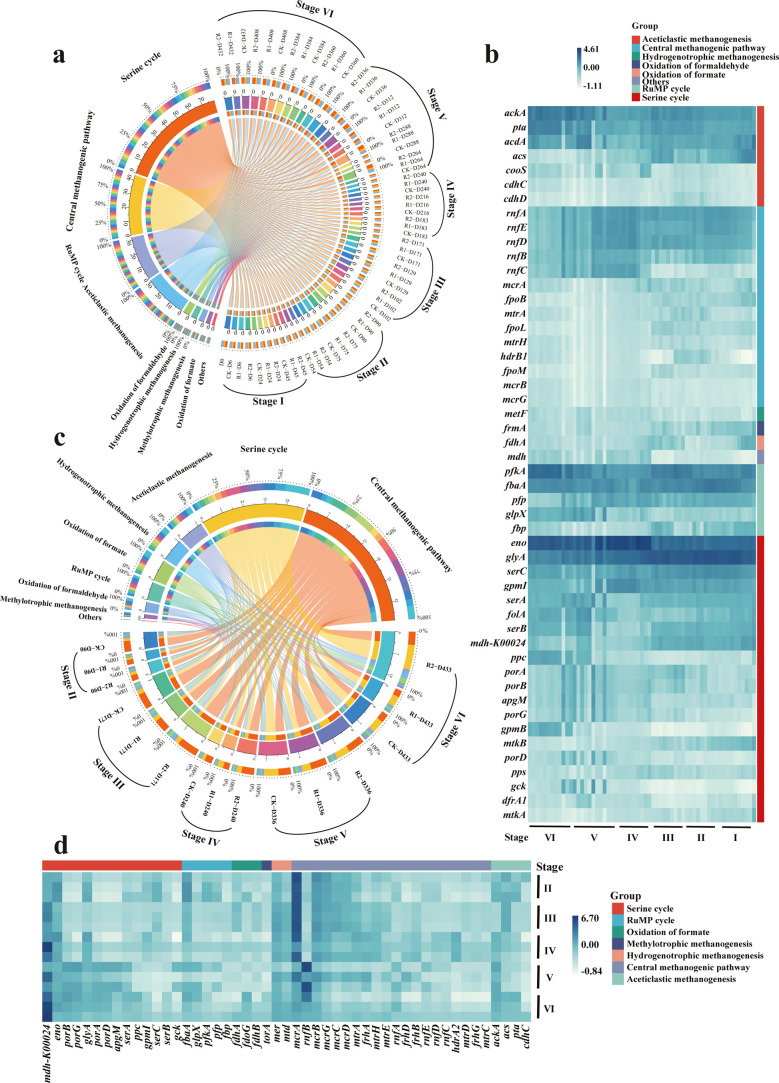
Dynamics of methane metabolism genes in anaerobic digestion. (a) Relative abundance of methane metabolism (MM) functional modules (KEGG) across six operational stages. (b) Heatmap of individual MM gene relative abundances (DNA level, log-scaled RPKM). (c) Expression levels of MM functional modules (meta-transcriptomic RPKM) across stages. (d) Heatmap of MM gene expression (log-scaled RPKM). Color scales in (a) and (c) represent module-level relative abundance/expression; heatmaps (b,d) are z-score normalized per gene.

Meta-transcriptomic profiling revealed that the central methanogenic pathway dominated MM gene expression (42.0%), followed by the serine cycle (29.0%). Acetoclastic methanogenesis expression (7.0%) was comparable to hydrogenotrophic methanogenesis (6.9%), with minor contributions from formaldehyde oxidation (6.0%) and the RuMP cycle (5.0%) (Figure 2c). The *mcrA* gene exhibited the highest expression within the central methanogenic pathway, despite lower DNA-level abundance, highlighting its critical role in terminal methane formation (Figure 2d). SRT extension (Stage III) significantly upregulated central methanogenic pathway expression (Kruskal–Wallis test, *P* < 0.01), yet daily methane yields declined, further reinforcing substrate limitation as the key constraint. Thermophilic conditions (Stage IV) suppressed expression of the central methanogenic pathway and acetoclastic methanogenesis (Kruskal–Wallis test, *P* < 0.001), mirroring DNA-level reductions and correlating with the thermophilic inhibition. Increasing TS to 15% and 20% enhanced expression of upstream modules, including *rnfB* in the central pathway and *ackA*/*acs* in acetoclastic methanogenesis, but *mcrA* expression remained stable. This decoupling led to volatile fatty acids (VFAs) accumulation, as evidenced by a significant pH drop (from ∼7.5 to 6.5; Kruskal–Wallis test, *P* < 0.001) and sCOD increase in Stages V and VI, contributing to AD instability (Figure 1b,c). At Stage IV, elevated serine cycle expression, driven by mdh (K00024), which links carbon metabolism to redox balance via malate-to-oxaloacetate conversion, indicated a metabolic shift. Iron compounds did not significantly influence MM gene expression profiles, further suggesting their impact on methane production operates via microenvironmental factors (e.g., pH or electron transfer) or protein-level regulation rather than transcriptional changes. Collectively, *mcrA* abundance and expression patterns robustly explained daily methane production dynamics, with acetoclastic methanogenesis as the primary pathway and temperature/TS as key drivers of metabolic perturbations.

### Viral AMGs in AD

We identified viral sequences from metagenomic and meta-transcriptomic data at the contigs level. A total of 6,210 nonredundant DNA vOTUs were identified using a combination of VirSorter2, VirFinder, and geNomad, followed by manual curation with CheckV. Additionally, 517 RNA vOTUs were detected based on RNA-directed RNA polymerase (RdRp) homology and geNomad. We adopted an inclusive approach using DRAM-v and VIBRANT annotations to capture context-specific functions in AD, although there still exists debate on the AMG identification (see Supporting Information S2). Combined annotation revealed 1,194 AMGs across 861 vOTUs, with an average of 184.1 ± 38.8 AMGs per sample (Table S2). AMGs were predominantly associated with amino acid metabolism (43.0%), followed by cofactor and vitamin metabolism (18.0%), energy metabolism (10.0%), and carbohydrate metabolism (9.0%) (Figure 3a). Notably, 10 vOTUs carried six AMGs directly linked to MM: *serA* (K00058), *glyA* (K00600), *eno* (K01689), *gck* (K11529), *gpml* (K15633), and *fwdH* (K00204). These MM AMGs primarily contributed to the serine cycle, except for *fwdH*, which supports hydrogenotrophic methanogenesis. These MM AMGs were also observed in recent studies on methane-rich environments.^2,51^ Among non-MM AMGs, *dcm* (K00558; DNA cytosine-5-methyltransferase) and *metK* (K00789; S-adenosylmethionine synthetase) were most prevalent (Figure 3c), although their classification as AMGs is debated due to potential roles in viral replication or host defense rather than host metabolic augmentation (see Supplementary discussion).^42^ In contrast, only one RNA vOTU (vOTU473) carried an AMG (K12448; UDP-arabinose 4-epimerase) linked to amino sugar metabolism, with no MM AMGs detected, highlighting distinct contributions of DNA and RNA phages.

**Figure 3:**
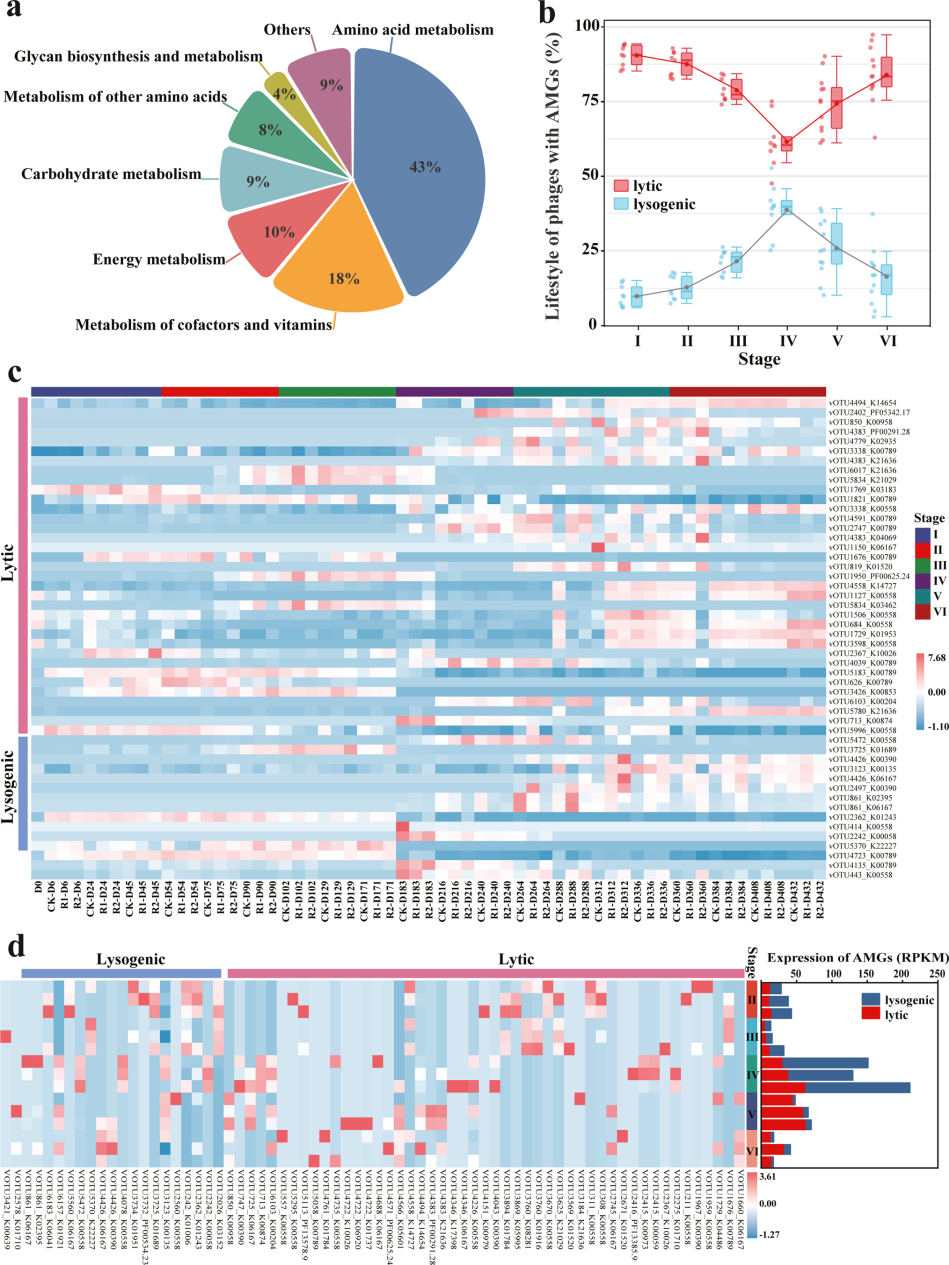
Composition and dynamics of viral auxiliary metabolic genes (AMGs) in anaerobic digestion. (a) Functional classification of the identified AMGs. (b) Proportion of lytic vs lysogenic phages carrying AMGs across operational stages. (c) Heatmap of AMG relative abundance at the DNA level (log_2_ RPKM, z-score normalized per gene). (d) Heatmap of AMG expression (meta-transcriptomic log_2_ RPKM, z-score normalized per gene); bar plot (right) shows total AMG expression and the contribution of lysogenic (blue) vs lytic (red) vOTUs per stage.

Operational parameters, particularly temperature and TS, significantly influenced AMG composition and lifestyle at the DNA abundance level (Figure 3b). At Stages I–II (mesophilic, 35 °C), AMGs were primarily associated with lytic phages (77.2% of total AMG abundance), with lytic vOTU176_K03183 (*ubiE*, coenzyme Q biosynthesis) dominating. Extending SRT to 24 days (Stage III) doubled lysogenic AMG abundance to 21.4%, with lysogenic vOTU3725_K01689 (*eno*, serine cycle) becoming prominent, suggesting AMGs aided host survival under substrate limitation, although lytic AMGs still dominated overall abundance. Transition to thermophilic conditions (55 °C, Stage IV) increased lysogenic AMG abundance to 38.7%, with lysogenic vOTU224_K00058 (*serA*) and vOTU5472_K00558 (*dcm*) dominating. As directly confirmed by our Hi-C infection network (detailed in the subsequent section), these dominant lysogenic phages were actively infecting the dominant microbial hosts (e.g., MAG37), reflecting “piggyback-the-winner” dynamics to enhance host adaptation to thermal stress, consistent with reduced MM gene abundances and expression. Increasing TS to 15% and 20% (Stages V–VI) shifted AMG composition toward lytic AMGs, such as vOTU4494_K14654 (*arfC*, nucleotide metabolism). During these stages, the reactors experienced a metabolic imbalance where fast-growing fermentative bacteria overproduced VFAs, leading to VFA accumulation and a significant pH decline. We hypothesize that the enriched lytic AMGs facilitated rapid phage replication to actively lyse these overproliferating VFA producers. Ecologically, this represents a “kill-the-winner” dynamic, acting as a top-down control mechanism that attempts to curb VFA overproduction and restore system equilibrium.

Meta-transcriptomic analysis revealed that only 14.5% (173/1193) of AMGs were actively expressed, with lysogenic AMGs often dominating expression despite lytic AMGs frequently prevailing in DNA abundance, highlighting differential activity under operational stress. Mantel test showed that AMG expression was closely associated with methane production (rho = 0.3849, *P* < 0.001, permutations = 999), indicating the important role of AMGs in MM. At Stages II–III, although lytic AMGs dominated DNA abundance (∼78.6%), lysogenic AMGs accounted for 62.4% of the expression, led by vOTU242_K01006 (*ppdK*, carbon flux regulation), vOTU3725_K01689 (*eno*, serine cycle), and vOTU2026_K03152 (protein deglycase, stress protection) (Figure 3d). These expressed AMGs likely conferred competitive advantages to hosts by supporting energy-efficient carbon metabolism (*ppdK*), serine cycle activity (*eno*), and cellular defense against oxidative stress (K03152), indirectly sustaining methanogenesis via “piggyback-the-winner” dynamics, even as substrate limitation reduced daily methane yields. Under thermophilic conditions (Stage IV), lysogenic AMG expression peaked at 73.2%, aligning with increased lysogenic abundance at the DNA level (38.7%), with vOTU5472_K00558 (*dcm*) and vOTU4078_K00558 dominating, potentially aiding host adaptation to thermal stress through methylation-mediated protection, though not fully restoring daily methane production. In contrast, increasing TS to 15% and 20% (Stages V–VI) reduced lysogenic AMG expression to 13.6%, while lytic AMGs dominated (80.7%), consistent with their enriched DNA abundance, such as vOTU850_K00958 (*sat*, sulfate reduction), reflecting a shift to “kill-the-winner” dynamics to regulate microbial overgrowth of the fast-growing bacteria amid high organic loading. This coincided with VFA accumulation reflected by pH decline and reduced daily methane yields (Figure 1a), suggesting expressed lytic AMGs attempted to restore system stability by curbing fast-growing populations.

In summary, we think lysogenic AMGs dominated regulation in Stages II–IV by enhancing host resilience via “piggyback-the-winner” dynamics, stabilizing methane production under substrate limitation and thermal stress. However, at Stages V–VI, increased TS disrupted this balance between VFA generation and methane production, reflected by the pH decline, with lytic AMGs prevailing to restore system stability through “kill-the-winner” dynamics targeting fast-growing bacteria.

### Virus–Host Interactions in AD

Hi-C identified 6100 unique phage–host interactions, linking 1817 DNA vOTUs, 5 RNA vOTUs, and 253 MAGs, covering 84.3% of detected MAGs and 60.0% of vOTUs per stage, demonstrating robust representation of AD phage–host interactions. Bacillota-dominated phage infections (70.0%) were followed by Archaea (10.1%) and Bacteroidota (8.0%) (Figure 4). Mantel test and Procrustes analysis revealed that viral communities explained 93.2% of bacterial community variation (rho = 0.986, *P* < 0.0001), despite 90% of vOTUs lacking family-level taxonomic assignment and only 23.7% of viral open reading frames (vORFs) finding homologues in NCBI Virus RefSeq (74.5% in IMG/VR 3.0), indicating a largely uncharacterized viral diversity in AD. Viral communities also showed significant correlation with methane production (Mantel test, ρ = 0.2664, *P* < 0.001, permutations = 999). These findings underscore the important role of phages in modulating the microbial communities responsible for methane production.

**Figure 4:**
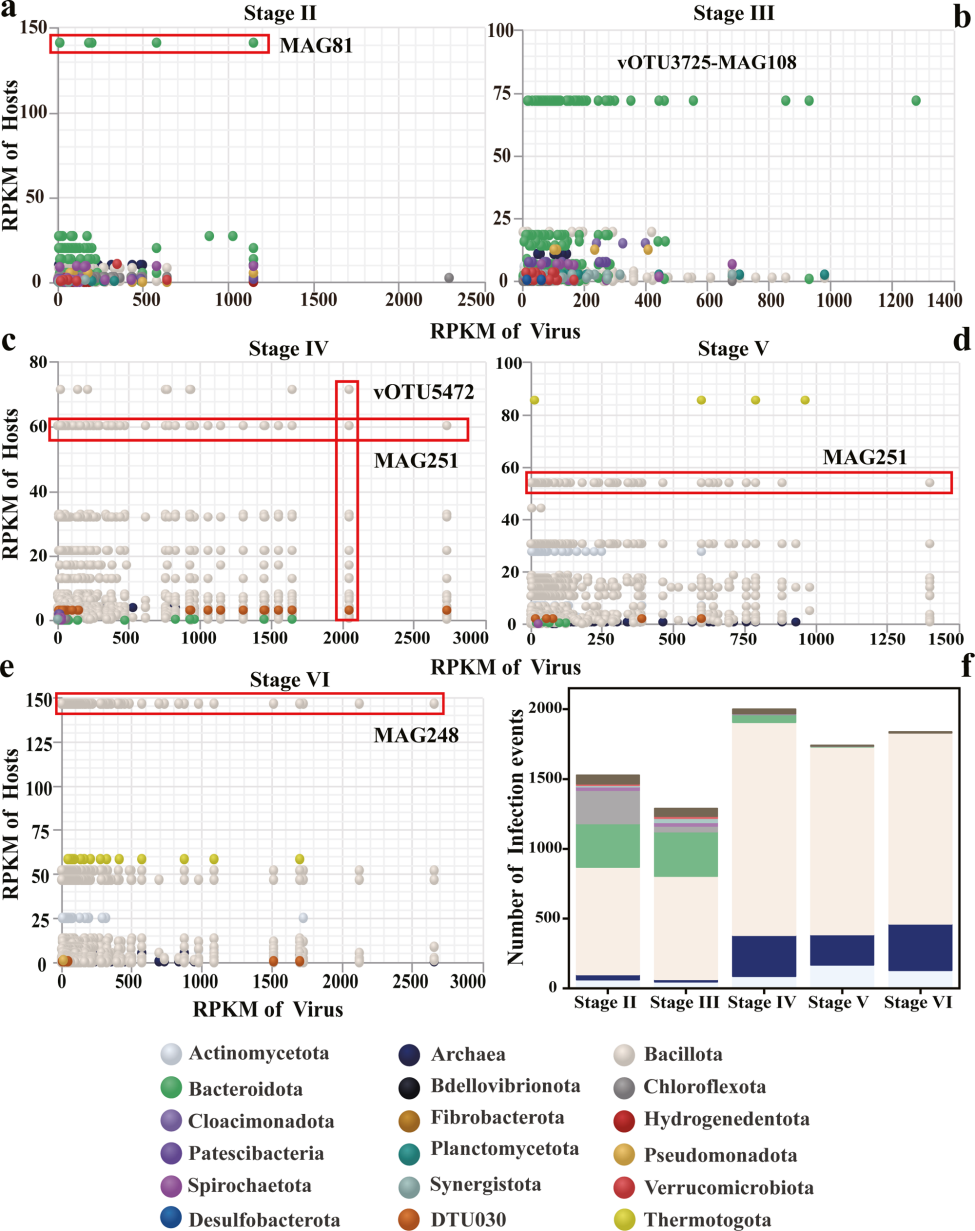
The ongoing virus–host interactions revealed by Hi-C. (a–e) The connection between vOTU and MAG revealed by Hi-C concerning the relative abundance (RPKM) within each stage (II–VI). Each point represents a single infection event revealed by Hi-C (≥5 contacts). (f) Total number of Hi-C-supported infection events per stage and taxonomic distribution of infected MAGs at the phylum level. Point and bar-plot colors denote MAG phyla (legend).

Operational parameters significantly altered phage–host interaction dynamics (Figure 4a–e). At Stages I–II (mesophilic, 35 °C), Bacillota infections dominated (53.8%), despite Bacteroidota comprising a higher abundance (e.g., 53.8% at Stage II, Figure S2). Bacillota experienced twice as many phage infections as Bacteroidota (22.6%), suggesting their greater susceptibility in AD. Extending SRT to 24 days (Stage III) reduced Chloroflexota infections, correlating with their abundance decline (0.6% to 0.1%). Transition to thermophilic conditions (55 °C, Stage IV) markedly reduced Bacteroidota infections (<1.0%) while further increasing Bacillota infections (75.8%) and methanogen infections (from 1.9% to 15.1%; Kruskal–Wallis test, *P* < 0.001, Figure 4f). Total phage–host interactions increased from 1,408 to 1,859 in Stage IV, despite stable node counts (∼720), indicating enhanced broad-host-range phage behavior. At mesophilic conditions, specific vOTUs (infecting one MAG) dominated (50% of vOTUs), but at thermophilic conditions, broad-host-range vOTUs prevailed (80.0% of relative abundance vs 50% at mesophilic; Kruskal–Wallis test, *P* < 0.01). We can also see that the dominant vOTUs are specific at mesophilic conditions, but the broad-host-range vOTUs became dominant at thermophilic conditions (Figure 4a–e). The dominant MAGs also experienced much more vOTU infections at thermophilic conditions like the MAG251 (96 vOTUs) and MAG248 (120 vOTUs). The average number of infecting vOTUs per MAG doubled from 11.2 (mesophilic) to 23.7 (thermophilic), reflecting stronger phage selective pressure. The phage lifestyle analysis indicated 69.5 ± 6.6% of all the vOTUs were lytic, and the ongoing lytic infections also dominated (90.2%) over lysogenic (9.8%), but they both showed no significant parameter-driven shifts.

Focusing on methanogens, 581 phage–host interactions involved 9 of 10 methanogenic MAGs, encompassing 529 DNA vOTUs and 4 RNA vOTUs. Notably, 488 vOTUs infected MAG37 (*Methanoculleus thermophilus*) exclusively under thermophilic conditions, including four RNA vOTUs (vOTU167, vOTU191, vOTU301, and vOTU325), marking the first reported RNA phage infections of methanogens (Figure 5a). Phylogenetic analysis of RNA-dependent RNA polymerase (RdRp) classified vOTU167 as an ssRNA phage related to Chimpavirus luticola (activated sludge origin), infecting MAG37, MAG127, MAG38, and MAG51 (Figure 5a). The other three (vOTU191, vOTU301, and vOTU325) aligned with eukaryotic viruses (Hubei picorna-like virus 61, Picobirnavirus dog/KNA/2015, and Antonospora locustae virus 1), with vOTU191 infecting nine additional MAGs and vOTU301 infecting MAG141. Broad-host-range infections were prevalent, with 438 of 533 methanogen-infecting vOTUs targeting both archaea and bacteria across multiple phyla. For example, vOTU968 infected 51 MAGs, spanning methanogens and six bacterial phyla. These cross-domain infections are widely spread and highlight the ecological versatility of phages in AD. Phages infecting methanogens may have evolved from bacterial or eukaryotic viruses or *vice versa*, reflecting the unique evolutionary and infection dynamics of methanogens. The integration of Hi-C with metagenomics and meta-transcriptomics enabled the discovery of novel DNA and RNA phages, particularly those targeting hard-to-culture methanogens, expanding our understanding of the viral diversity in AD.

**Figure 5:**
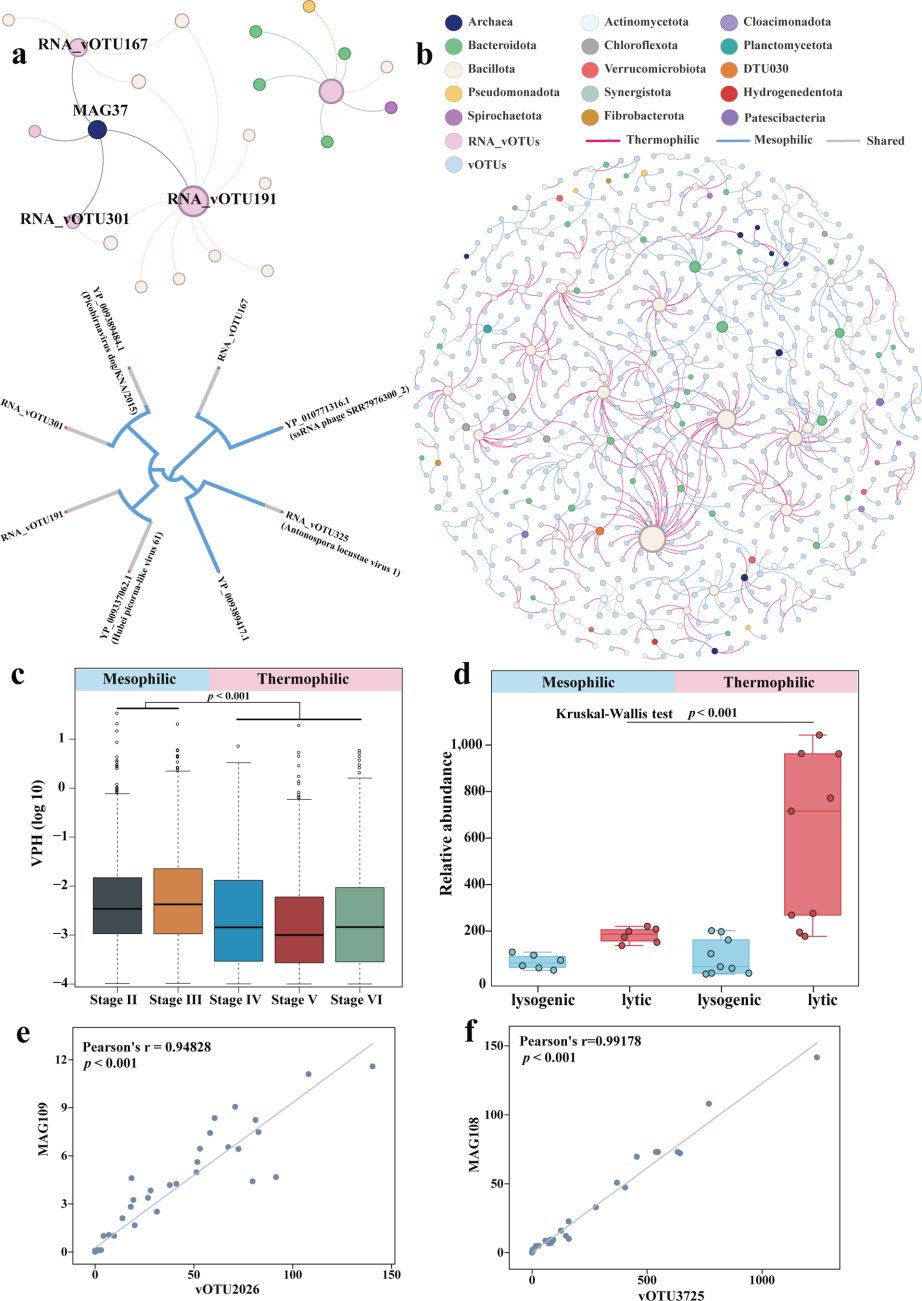
RNA phages, historical linkages, and active infection dynamics revealed by Hi-C. (a) Network and RdRp-based phylogeny of the four RNA vOTUs infecting methanogens (exclusively under thermophilic conditions; first reported archaeal RNA phage infections). (b) Historical virus–host interaction network inferred from CRISPR spacers, tRNAs, and sequence homology. (c) Viral copies per host cell (VPH) across operational stages. (d) Expression (RPKM) of lysogenic vs lytic vOTUs mediating active Hi-C linkages under mesophilic vs thermophilic conditions (Kruskal–Wallis test, *P* < 0.01). (e,f) Significantly positive correlations between the relative abundance of the vOTUs with AMGs and their connected hosts (Pearson correlation, vOTU2026-MAG109, *r* = 0.948; vOTU3725-MAG108, *r* = 0.992; both *P* < 0.001).

To validate active infections, we identified 702 historical phage–host interactions via CRISPR-Cas spacer, tRNA, and homology matching, with 429 (61.1%) overlapping Hi-C results (Figure 5b). These analyses identified infections involving 15 additional MAGs, with 67.9% showing phage specificity and 78.7% involving lytic phages (21.3% are lysogenic). Notably, 9 vOTUs infected 7 methanogenic MAGs, reinforcing Hi-C findings of enhanced methanogen infections at thermophilic conditions. These historical traces corroborate the active, broad-host-range phage interactions observed *via* Hi-C, particularly under thermal stress, underscoring phages’ important role in regulating methanogenic communities and methane production in AD.

### Viral Modulation of MM in AD

To investigate phage-mediated modulation of MM in AD, we analyzed the transcriptional activity of vOTUs integrated with Hi-C-derived phage–host interactions. At thermophilic conditions (Stage IV, 55 °C), active phage–host interactions increased, but viral copies per host (VPH) decreased significantly from 0.09 to 0.04 (Figure 5c). Concurrently, viral transcriptional activity surged from 219.1 to 1081.6 RPKM (Kruskal–Wallis test, *P* < 0.001) at Stage IV, with lytic vOTUs dominating expression (71.7%), although the ratio of lytic (90.2%) vs lysogenic (9.8%) vOTUs changed little at the DNA level. In contrast, mesophilic conditions favored lysogenic infections, with lower lytic vOTU expression (40.3%) and higher VPH, reflecting “piggyback-the-winner” dynamics that stabilize methane production (Figure 5d). With increasing TS to 15% and 20% (Stages V–VI), transcriptional activity declined to 696.9 and 266.3 RPKM, respectively (Kruskal–Wallis test, *P* < 0.001), yet lytic vOTU expression still increased to 71.7% at thermophilic conditions (Figure 5d, Kruskal–Wallis test, *P* < 0.01), further suggesting intensified “kill-the-winner” efforts to restore microbial balance amid system instability.

Based on a stringent dual-pipeline identification approach (detailed in Methods and Text S2), we observed that AMGs played a pivotal role in phage–host interactions, with 16.3 ± 2.1% of interactions linked to AMGs across all stages. At Stages II–III (mesophilic), lysogenic AMGs dominated expression, including vOTU3725_K01689, vOTU242_K01006, and vOTU2026_K03152, accounting for 62.4% of AMG expression (Figure 3d). Hi-C revealed that vOTU3725 (*Aliceevansviridae*) and vOTU2026 (Caudoviricetes) targeted MAG108 (Bacteroidota_DMER64 sp001512865) and MAG109 (Bacteroidota_JAJQAW01), respectively, both dominant species at Stages II–III (Figure 4a,e). Correlation analysis showed a significantly positive relationship between vOTU3725 and MAG108 (Figure 5e, Pearson, *r* = 0.992, *P* < 0.01) and vOTU2026 and MAG109 (Figure 5f, Pearson, *r* = 0.948, *P* < 0.01). These indicated that AMGs enabled host dominance and stabilized methane production via “piggyback-the-winner” dynamics. At Stage IV (thermophilic, 55 °C), lysogenic AMG expression peaked at 73.2%, driven by vOTU5472 (Caudoviricetes) carrying *dcm* (K00558, DNA methyltransferase), which also dominated DNA-level abundance (Figures 3d and 4c). Hi-C identified vOTU5472 as a broad-host-range phage infecting 23 MAGs, including *M. thermophilus* (MAG37) and bacteria across multiple phyla. Of these, 22 MAGs showed significant positive correlations with vOTU5472 abundance (Pearson, *r* > 0.85, *P* < 0.01), and most of them were dominant species absent in mesophilic stages, indicating *dcm* enhanced host genome stability and metabolic adaptability under thermal stress, supporting methanogenesis via piggyback-the-winner dynamics. However, increasing TS to 15% and 20% (Stages V–VI) shifted AMG expression to lytic vOTUs (80.7%), with vOTU850 (*Podoviridae*, K00958, *sat*, sulfate adenylyltransferase) dominating (Figure 3c). Hi-C and sequence-based analyses failed to identify vOTU850 hosts, suggesting *sat* supported rapid phage replication rather than host competitive advantage. We think this shift to kill-the-winner dynamics coincided with VFAs accumulation reflected by pH decline and reduced methane yields, indicating lytic phages attempted to restore microbial balance by targeting fast-growing populations as the VFA producers. For MM-specific AMGs, *fwdH* (K00204, formate dehydrogenase) on lytic vOTU6103 was silent at mesophilic conditions but expressed at Stage IV. *fwdH* catalyzes formic acid oxidation to CO_2_ with NADH generation, potentially boosting host redox balance and C1 metabolism. However, we think its expression primarily might support rapid phage replication rather than enhanced methanogenesis.

Collectively, lysogenic AMGs (*eno*, *ppdK*, and *dcm*) facilitated host dominance and methane production stability in Stages II–IV, while lytic AMGs (*sat*) in Stages V–VI prioritized phage replication, reflecting a shift from stabilizing to disruptive viral modulation under high organic loading. These findings highlight the context-specific roles of AMGs in regulating methanogenic communities and methane production in AD.

## Discussion

By integrating Hi-C with metagenomics and meta-transcriptomics, we elucidated the dynamic role of DNA and RNA phages in modulating methane production in AD through an over 440 day semi-continuous experiment. Our findings reveal how operational parameters, including iron compounds amendment, temperature, SRT, and TS, shape phage–host interactions, with distinct ecological strategies governing microbial community stability and methane production.

### Virus–Host Interactions Underpin the Stability of Methane Production in AD

Two key ecological models, “piggyback-the-winner” and “kill-the-winner,” underpin these dynamics.^10^ In the “piggyback-the-winner” model, lysogenic phages integrate into host genomes, enhancing host fitness without immediate lysis, while “kill-the-winner” involves lytic phages targeting fast-growing bacteria as the fermenters to regulate community composition and resource availability. These strategies align with microbial ecology principles, where lysogenic phages foster mutualistic interactions under stable or stressful conditions, and lytic phages drive nutrient cycling and microbial diversity under nutrient-rich environments.^52^ During mesophilic conditions (Stages I–III, 35 °C), efficient organic matter utilization (low sCOD) coincided with lysogenic AMGs dominating expression (62.4%), despite higher lytic vOTU abundance. AMGs such as *eno* (K01689) and *ppdK* (K01006) enhanced host competitive advantages in Bacteroidota (e.g., MAG108 and MAG109), stabilizing methane production (681.6 ± 218.3 mL CH_4_ L^–1^) via “piggyback-the-winner” dynamics. Transition to thermophilic conditions (Stage IV, 55 °C) triggered a surge in viral transcriptional activity (1081.6 RPKM) and methanogen infections (15.1%), with lysogenic AMGs like *dcm* (K00558) peaking in expression (73.2%). These AMGs, carried by broad-host-range vOTUs (e.g., vOTU5472), supported host genome stability and metabolic adaptability in dominant species (e.g., *M. thermophilus*, MAG37), promoting adaptation of the system to thermophilic conditions. However, increasing TS to 15% and 20% (Stages V–VI) disrupted the balance between VFA production and methane formation, elevating VFA accumulation and reducing pH, leading to system instability. Lytic AMGs, such as *sat* (K00958), dominated expression (80.7%), prioritizing rapid phage replication over host fitness, as evidenced by the inability to identify vOTU850 hosts via Hi-C or CRISPR-Cas analyses. This shift to “kill-the-winner” dynamics aimed to curb fast-growing bacteria like the VFA producers to restore methane yields, as methanogenesis, the rate-limiting step, was outpaced by VFAs production.

### RNA Phages also Play an Important Role in AD

We also discovered four RNA phages (vOTU167, vOTU191, vOTU301, and vOTU325) that infect methanogens exclusively under thermophilic conditions, marking the first reported RNA phage–methanogen interactions.^53^ Phylogenetic analysis suggests that these RNA phages, some related to eukaryotic viruses (e.g., Hubei picorna-like virus 61), may have evolved from bacterial or eukaryotic viruses, or *vice versa*, highlighting the unique evolutionary dynamics of methanogens. The prevalence of broad-host-range phages, such as vOTU968 infecting 51 MAGs across six phyla, underscores the ecological versatility of phages in AD, potentially bridging bacterial and archaeal domains. The integration of Hi-C with multi-omics was critical to these discoveries, enabling the detection of novel DNA and RNA phages targeting hard-to-culture methanogens. However, Hi-C has its limitations, as it cannot distinguish whether DNA originates from active phage infection or horizontal gene transfer.^54^ Historical interaction traces overlapped 429 of Hi-C interactions, but uncertainties remain, warranting caution in interpreting single-cell-level data.

### Phage-Based Strategies for Bioenergy and Emission Mitigation

These findings reposition phages as important regulators of methane cycling, with dual roles in direct metabolic enhancement *via* AMGs and indirect community restructuring via lysis. From a quantitative perspective, our statistical modeling demonstrates that viral community shifts and AMG expressions are significantly correlated with macroscopic methane yield variations, underscoring their profound engineering relevance. Compared to conventional AD optimization strategies, such as the direct dosing of chemical agents (e.g., the iron amendments utilized in this study) or nonspecific bioaugmentation, phage-based biocontrol offers unprecedented target specificity without accumulating chemical residues in the digestate. For bioenergy applications, engineering phage communities to amplify lysogenic AMGs (e.g., *dcm* for thermal stability) or modulate lytic cycles could optimize methane yields. For instance, enriching phage suspensions with lysogenic AMGs or editing phages to carry stress-adaptive AMGs could enhance methanogen resilience under environmental stressors like temperature shocks. In high-TS conditions, targeted lytic phages could suppress fast-growing VFA producers, rebalancing methanogenesis, as attempted by vOTU850 in Stages V–VI. Nonetheless, from a methane emission control perspective, lysogenic AMGs enhance methanogenesis in nutrient-poor environments (e.g., Stages I–III, low sCOD due to efficient methane production), suggesting that lytic phages targeting methanogens could reduce emissions in systems like ruminant guts, where cellulose hard-to-degradation creates a nutrient-limited state.^55^ However, this risks acid accumulation, potentially problematic in ruminant systems but viable in sediments or rivers. In nutrient-rich environments like peatlands,^56^ lytic phages targeting VFA producers could limit substrate availability for methanogenesis, offering a novel emission control strategy.

Nonetheless, it is important to acknowledge that our current findings rely fundamentally on omics-based inference. While the integration of Hi-C and meta-transcriptomics provides robust in situ evidence of physical phage–host linkages and active transcriptional responses, establishing definitive causality requires direct experimental validation. Furthermore, translating these conceptual strategies into operational feasibility on a full-scale bioreactor level faces substantial engineering challenges. Current limitations include the difficulties in the pure-culture isolation of uncultured phages, the techno-economic costs of large-scale phage mass production, and the potential for hosts to rapidly develop an antiviral defense system. To mechanistically confirm the precise pathways of phage-mediated metabolic regulation, future research should move toward experimental validation. However, we acknowledge that the pure-culture isolation of specific methanogenic archaea and their phages presents formidable technical challenges, primarily due to their stringent anaerobic requirements, slow growth rates, and obligate syntrophic dependencies.^57^ Therefore, as a highly feasible intermediate step, future lab-scale validation experiments could prioritize targeted enrichment cultures. Coupling these bioreactor enrichments with stable isotope probing or single-cell viromics could practically track viral infection dynamics and metabolic fluxes in vitro before attempting pure-culture isolation. In addition, future studies should continue to leverage single-cell Hi-C and meta-transcriptomics to quantify AMG contributions and validate RNA phage roles across diverse methanogenic environments. Exploring AMG evolution under varying viral lifestyles and environmental stressors will further elucidate its ecological functions. Ultimately, we believe that these insights provide a foundational framework for developing phage-based strategies to optimize bioenergy production and mitigate greenhouse gas emissions, advancing our understanding of viral contributions to global carbon dynamics.

## Supplementary Material





## Data Availability

The raw metagenomic sequence data generated in this study are deposited in NGDC (National Genomics Data Center, https://ngdc.cncb.ac.cn/) with accession number CRA013482. The assembled MAGs, DNA vOTUs, and RNA vOTUs have been deposited into FigShare (10.6084/m9.figshare.32324328).

## Notes

Identification of RNA vOTUs along with ARGs and host linkage analysis; auxiliary metabolic genes identification; changes of bacterial community composition and activity in AD; changes in viral community in AD; mapped methane metabolism genes from the collected MAGs in the KEGG methane metabolism (map00680); changes in microbial community at the phylum level (a) and heatmap showing the changes of microbial community at species level (b) along with anaerobic digestion; and procrustes analysis showing the significant correlation between viral and bacterial community in anaerobic digestion (PDF)
The deposition information on the sequenced samples in this study; detailed information on the collected MAGs; and detailed information on the collected AMGs (XLSX)

## References

[1] IPCC . Climate Change 2023: Synthesis Report; 2023.

[2] Zhong Z. P., Du J., Köstlbacher S., Pjevac P., Orlić S., Sullivan M. B. (2024). Viral Potential
to Modulate Microbial Methane Metabolism
Varies by Habitat. Nat. Commun..

[3] Conrad R. (2009). The Global
Methane Cycle: Recent Advances in Understanding the Microbial Processes
Involved. Environ. Microbiol. Rep..

[4] Dueholm M. K. D., Andersen K. S., Korntved A. K. C., Rudkjøbing V., Alves M., Bajón-Fernández Y., Batstone D., Butler C., Cruz M. C., Davidsson Å., Erijman L., Holliger C., Koch K., Kreuzinger N., Lee C., Lyberatos G., Mutnuri S., O’Flaherty V., Oleskowicz-Popiel P., Pokorna D., Rajal V., Recktenwald M., Rodríguez J., Saikaly P. E., Tooker N., Vierheilig J., De Vrieze J., Wurzbacher C., Nielsen P. H. (2024). MiDAS 5: Global
Diversity of Bacteria and Archaea in Anaerobic Digesters. Nat. Commun..

[5] Borrel G., Adam P. S., McKay L. J., Chen L. X., Sierra-García I. N., Sieber C. M. K., Letourneur Q., Ghozlane A., Andersen G. L., Li W. J., Hallam S. J., Muyzer G., de Oliveira V. M., Inskeep W. P., Banfield J. F., Gribaldo S. (2019). Wide Diversity of Methane
and Short-Chain Alkane Metabolisms in Uncultured Archaea. Nat. Microbiol..

[6] Evans P. N., Boyd J. A., Leu A. O., Woodcroft B. J., Parks D. H., Hugenholtz P., Tyson G. W. (2019). An Evolving View
of Methane Metabolism in the Archaea. Nat. Rev.
Microbiol..

[7] Jassey A., Jackson W. T. (2024). Viruses and Autophagy:
Bend, but Don’t Break. Nat. Rev. Microbiol..

[8] Carreira C., Lønborg C., Acharya B., Aryal L., Buivydaite Z., Borim Corrêa F., Chen T., Lorenzen Elberg C., Emerson J. B., Hillary L., Khadka R. B., Langlois V., Mason-Jones K., Netherway T., Sutela S., Trubl G., wa Kang’eri A., Wang R., White R. A., Winding A., Zhao T., Sapkota R. (2024). Integrating Viruses into Soil Food
Web Biogeochemistry. Nat. Microbiol..

[9] Ma B., Wang Y., Zhao K., Stirling E., Lv X., Yu Y., Hu L., Tang C., Wu C., Dong B., Xue R., Dahlgren R. A., Tan X., Dai H., Zhu Y. G., Chu H., Xu J. (2024). Biogeographic Patterns and Drivers of Soil Viromes. Nat. Ecol. Evol..

[10] Chen X., Weinbauer M. G., Jiao N., Zhang R. (2021). Revisiting
Marine Lytic
and Lysogenic Virus-Host Interactions: Kill-the-Winner and Piggyback-the-Winner. Sci. Bull..

[11] Huang L., Yang B., Yi H., Asif A., Wang J., Lithgow T., Zhang H., Minhas F., Yin Y. (2021). AcrDB: A Database
of Anti-CRISPR Operons in Prokaryotes and Viruses. Nucleic Acids Res..

[12] Prangishvili D., Forterre P., Garrett R. A. (2006). Viruses of the Archaea: A Unifying
View. Nat. Rev. Microbiol..

[13] Li R., Wang Y., Hu H., Tan Y., Ma Y. (2022). Metagenomic
Analysis Reveals Unexplored Diversity of Archaeal Virome in the Human
Gut. Nat. Commun..

[14] Yaffe E., Relman D. A. (2020). Tracking Microbial Evolution in the Human Gut Using
Hi-C Reveals Extensive Horizontal Gene Transfer, Persistence and Adaptation. Nat. Microbiol..

[15] Wu R., Davison M. R., Nelson W. C., Smith M. L., Lipton M. S., Jansson J. K., McClure R. S., McDermott J. E., Hofmockel K. S. (2023). Hi-C Metagenome Sequencing Reveals Soil Phage–Host
Interactions. Nat. Commun..

[16] International Energy Agency . Outlook for Biogas and Biomethane; 2020..

[17] Vasco-Correa J., Khanal S., Manandhar A., Shah A. (2018). Anaerobic Digestion
for Bioenergy Production: Global Status, Environmental and Techno-Economic
Implications, and Government Policies. Bioresour.
Technol..

[18] Du Y., Fuhrman J. A., Sun F. (2023). ViralCC Retrieves
Complete Viral
Genomes and Virus-Host Pairs from Metagenomic Hi-C Data. Nat. Commun..

[19] Zhang J., Lu T., Xin Y., Wei Y. (2022). Ferric Chloride Further Simplified
the Horizontal Gene Transfer Network of Antibiotic Resistance Genes
in Anaerobic Digestion. Sci. Total Environ..

[20] Lu T., Zhang J., Wei Y., Shen P. (2019). Effects of Ferric Oxide
on the Microbial Community and Functioning during Anaerobic Digestion
of Swine Manure. Bioresour. Technol..

[21] Uritskiy G. V., DiRuggiero J., Taylor J. (2018). MetaWRAP - A Flexible Pipeline for
Genome-Resolved Metagenomic Data Analysis. Microbiome.

[22] Qian L., Yu X., Zhou J., Gu H., Ding J., Peng Y., He Q., Tian Y., Liu J., Wang S., Wang C., Shu L., Yan Q., He J., Liu G., Tu Q., He Z. (2022). MCycDB: A Curated Database
for Comprehensively Profiling Methane
Cycling Processes of Environmental Microbiomes. Mol. Ecol. Resour..

[23] Olm M. R., Brown C. T., Brooks B., Banfield J. F. (2017). DRep: A Tool for
Fast and Accurate Genomic Comparisons That Enables Improved Genome
Recovery from Metagenomes through de-Replication. ISME J..

[24] Nayfach S., Shi Z. J., Seshadri R., Pollard K. S., Kyrpides N. C. (2019). New Insights
from Uncultivated Genomes of the Global Human Gut Microbiome. Nature.

[25] Bowers R. M., Kyrpides N. C., Stepanauskas R., Harmon-Smith M., Doud D., Reddy T. B. K., Schulz F., Jarett J., Rivers A. R., Eloe-Fadrosh E. A., Tringe S. G., Ivanova N. N., Copeland A., Clum A., Becraft E. D., Malmstrom R. R., Birren B., Podar M., Bork P., Weinstock G. M., Garrity G. M., Dodsworth J. A., Yooseph S., Sutton G., Glöckner F. O., Gilbert J. A., Nelson W. C., Hallam S. J., Jungbluth S. P., Ettema T. J. G., Tighe S., Konstantinidis K. T., Liu W. T., Baker B. J., Rattei T., Eisen J. A., Hedlund B., McMahon K. D., Fierer N., Knight R., Finn R., Cochrane G., Karsch-Mizrachi I., Tyson G. W., Rinke C., Lapidus A., Meyer F., Yilmaz P., Parks D. H., Murat Eren A., Schriml L., Banfield J. F., Hugenholtz P., Woyke T. (2017). Minimum Information about a Single Amplified Genome (MISAG) and a
Metagenome-Assembled Genome (MIMAG) of Bacteria and Archaea. Nat. Biotechnol..

[26] Chaumeil P. A., Mussig A. J., Hugenholtz P., Parks D. H. (2020). GTDB-Tk: A Toolkit
to Classify Genomes with the Genome Taxonomy Database. Bioinformatics.

[27] Shaffer M., Borton M. A., Mcgivern B. B., Zayed A. A., La Rosa S., Solden L. M., Liu P., Narrowe A. B., Rodríguez-Ramos J., Bolduc B., Gazitúa M. C., Daly R. A., Smith G. J., Vik D. R., Pope P. B., Sullivan M. B., Roux S., Wrighton K. C. (2020). DRAM for Distilling
Microbial Metabolism to Automate
the Curation of Microbiome Function. Nucleic
Acids Res..

[28] Zhang J., Lu T., Song Y., Rocha U. N. da, Liu J., Nikolausz M., Wei Y., Richnow H. H. (2024). Viral Communities Contribute More to the Lysis of Antibiotic-Resistant
Bacteria than the Transduction of Antibiotic Resistance Genes in Anaerobic
Digestion Revealed by Metagenomics. Environ.
Sci. Technol..

[29] Guo J., Bolduc B., Zayed A. A., Varsani A., Dominguez-huerta G., Delmont T. O., Pratama A. A., Gazitúa M. C., Vik D., Sullivan M. B., Roux S. (2021). VirSorter2: A Multi-Classifier, Expert-Guided
Approach to Detect Diverse DNA and RNA Viruses. Microbiome.

[30] Ren J., Ahlgren N. A., Lu Y. Y., Fuhrman J. A., Sun F. (2017). VirFinder:
A Novel k-Mer Based Tool for Identifying Viral Sequences from Assembled
Metagenomic Data. Microbiome.

[31] Camargo A. P., Roux S., Schulz F., Babinski M., Xu Y., Hu B., Chain P. S. G., Nayfach S., Kyrpides N. C. (2024). Identification of
Mobile Genetic Elements with GeNomad. Nat. Biotechnol..

[32] Roux S., Adriaenssens E. M., Dutilh B. E., Koonin E. V., Kropinski A. M., Krupovic M., Kuhn J. H., Lavigne R., Brister J. R., Varsani A., Amid C., Aziz R. K., Bordenstein S. R., Bork P., Breitbart M., Cochrane G. R., Daly R. A., Desnues C., Duhaime M. B., Emerson J. B., Enault F., Fuhrman J. A., Hingamp P., Hugenholtz P., Hurwitz B. L., Ivanova N. N., Labonté J. M., Lee K. B., Malmstrom R. R., Martinez-garcia M., Mizrachi I. K., Ogata H., Páez-Espino D., Petit M. A., Putonti C., Rattei T., Reyes A., Rodriguez-Valera F., Rosario K., Schriml L., Schulz F., Steward G. F., Sullivan M. B., Sunagawa S., Suttle C. A., Temperton B., Tringe S. G., Thurber R. V., Webster N. S., Whiteson K. L., Wilhelm S. W., Wommack K. E., Woyke T., Wrighton K. C., Yilmaz P., Yoshida T., Young M. J., Yutin N., Allen L. Z., Kyrpides N. C., Eloe-Fadrosh E. A. (2019). Minimum
Information about an Uncultivated Virus Genome (MIUVIG). Nat. Biotechnol..

[33] Bin
Jang H., Bolduc B., Zablocki O., Kuhn J. H., Roux S., Adriaenssens E. M., Brister J. R., Kropinski A. M., Krupovic M., Lavigne R., Turner D., Sullivan M. B. (2019). Taxonomic
Assignment of Uncultivated Prokaryotic Virus Genomes Is Enabled by
Gene-Sharing Networks. Nat. Biotechnol..

[34] Zheng X., Jahn M. T., Sun M., Friman V. P., Balcazar J. L., Wang J., Shi Y., Gong X., Hu F., Zhu Y. G., Zhu Y. G. (2022). Organochlorine
Contamination Enriches
Virus-Encoded Metabolism and Pesticide Degradation Associated Auxiliary
Genes in Soil Microbiomes. ISME J..

[35] Gregory A. C., Zablocki O., Zayed A. A., Howell A., Bolduc B., Sullivan M. B., Gregory A. C., Zablocki O., Zayed A. A., Howell A., Bolduc B. (2020). The Gut Virome
Database Reveals Age-Dependent
Patterns of Virome Diversity in the Human Gut. Cell Host Microbe.

[36] von
Meijenfeldt F. A. B., Arkhipova K., Cambuy D. D., Coutinho F. H., Dutilh B. E., Von Meijenfeldt F. A.
B., Arkhipova K., Cambuy D. D., Coutinho F. H., Dutilh B. E. (2019). Robust Taxonomic
Classification of Uncharted Microbial Sequences and Bins with CAT
and BAT. Genome Biol..

[37] Nayfach S., Camargo A. P., Schulz F., Eloe-Fadrosh E., Roux S., Kyrpides N. C. (2021). CheckV Assesses the Quality and Completeness
of Metagenome-Assembled Viral Genomes. Nat.
Biotechnol..

[38] Kieft K., Zhou Z., Anantharaman K. (2020). VIBRANT: Automated
Recovery, Annotation
and Curation of Microbial Viruses, and Evaluation of Viral Community
Function from Genomic Sequences. Microbiome.

[39] Shang J., Peng C., Liao H., Tang X., Sun Y. P.B. O. X. (2023). A Web Server for Identifying and Characterizing Phage
Contigs in
Metagenomic Data. Bioinforma. Adv..

[40] Wang T., Zhang P., Anantharaman K., Wang H., Zhang H., Zhang M., Xu J. (2025). Metagenomic
Analysis Reveals How
Multiple Stressors Disrupt Virus–Host Interactions in Multi-Trophic
Freshwater Mesocosms. Nat. Commun..

[41] Zayed A. A., Wainaina J. M., Dominguez-Huerta G., Pelletier E., Guo J., Mohssen M., Tian F., Pratama A. A. A., Bolduc B., Zablocki O., Cronin D., Solden L., Delage E., Alberti A., Aury J.-M. M., Carradec Q., da Silva C., Labadie K., Poulain J., Ruscheweyh H. J., Salazar G., Shatoff E., Bundschuh R., Fredrick K., Kubatko L. S., Chaffron S., Culley A. I., Sunagawa S., Kuhn J. H., Wincker P., Sullivan M. B., Acinas S. G., Babin M. (2022). Cryptic and Abundant
Marine Viruses at the Evolutionary Origins of Earth’s RNA Virome. Science.

[42] Martin C., Emerson J. B., Roux S., Anantharaman K. A. (2025). Call for
Caution in the Biological Interpretation of Viral Auxiliary Metabolic
Genes. Nat. Microbiol..

[43] ter
Horst A. M., Santos-Medellín C., Sorensen J. W., Zinke L. A., Wilson R. M., Johnston E. R., Trubl G., Pett-Ridge J., Blazewicz S. J., Hanson P. J., Chanton J. P., Schadt C. W., Kostka J. E., Emerson J. B. (2022). Minnesota Peat Viromes
Reveal Terrestrial and Aquatic Niche Partitioning for Local and Global
Viral Populations. Microbiome.

[44] Jahn M. T., Lachnit T., Markert S. M., Stigloher C., Pita L., Ribes M., Dutilh B. E., Hentschel U. (2021). Lifestyle
of Sponge Symbiont Phages by Host Prediction and Correlative Microscopy. ISME J..

[45] Hwang Y., Roux S., Coclet C., Krause S. J. E., Girguis P. R. (2023). Viruses
Interact with Hosts That Span Distantly Related Microbial Domains
in Dense Hydrothermal Mats. Nat. Microbiol..

[46] Aroney S. T. N., Newell R. J. P., Nissen J. N., Camargo A. P., Tyson G. W., Woodcroft B. J. (2025). CoverM:
Read Alignment Statistics for Metagenomics. Bioinformatics.

[47] Zhang J., Sui Q., Zhong H., Meng X., Wang Z., Wang Y., Wei Y. (2018). Impacts of
Zero Valent Iron, Natural Zeolite and Dnase on the Fate
of Antibiotic Resistance Genes during Thermophilic and Mesophilic
Anaerobic Digestion of Swine Manure. Bioresour.
Technol..

[48] Zahedi S., Gros M., Petrovi M., Balcazar J. L., Pijuan M. (2022). Anaerobic
Treatment of Swine Manure under Mesophilic and Thermophilic Temperatures:
Fate of Veterinary Drugs and Resistance Genes. Sci. Total Environ..

[49] Rocamora I., Wagland S. T., Villa R., Simpson E. W., Fernández O., Bajón-Fernández Y. (2020). Dry Anaerobic
Digestion of Organic
Waste: A Review of Operational Parameters and Their Impact on Process
Performance. Bioresour. Technol..

[50] Kohtz A. J., Petrosian N., Krukenberg V., Jay Z. J., Pilhofer M., Hatzenpichler R. (2024). Cultivation
and Visualization of a Methanogen of the
Phylum Thermoproteota. Nature.

[51] Chen X. P., Zhu D., Liu S. Y., Sun M. M., Ye M., Wang L., Lin D., Zhang T. L., Rillig M. C., Zhu Y. G. (2025). Unique Plastisphere
Viromes with Habitat-Dependent Potential for Modulating Global Methane
Cycle. Nat. Commun..

[52] Huang D., Yu P., Ye M., Schwarz C., Jiang X., Alvarez P. J. J. (2021). Enhanced
Mutualistic Symbiosis between Soil Phages and Bacteria with Elevated
Chromium-Induced Environmental Stress. Microbiome.

[53] Mihara T., Nishimura Y., Shimizu Y., Nishiyama H., Yoshikawa G., Uehara H., Hingamp P., Goto S., Ogata H. (2016). Linking Virus Genomes with Host Taxonomy. Viruses.

[54] Roux S., Coclet C. (2026). Viromics Approaches for the Study of Viral Diversity
and Ecology in Microbiomes. Nat. Rev. Genet..

[55] Wu Y., Gao N., Sun C., Feng T., Liu Q., Chen W. H. (2024). A Compendium
of Ruminant Gastrointestinal Phage Genomes Revealed a Higher Proportion
of Lytic Phages than in Any Other Environments. Microbiome.

[56] Juottonen H., Hynninen A., Nieminen M., Tuomivirta T. T., Tuittila E.-S., Nousiainen H., Kell D. K., Yrjälä K., Tervahauta A., Fritze H. (2012). Methane-Cycling Microbial Communities
and Methane Emission in Natural and Restored Peatlands. Appl. Environ. Microbiol..

[57] Medvedeva S., Borrel G., Krupovic M., Gribaldo S. (2023). A Compendium of Viruses
from Methanogenic Archaea Reveals Their Diversity and Adaptations
to the Gut Environment. Nat. Microbiol..

