# Supplementary material for: Insights Gained by High-throughput Chromosome Conformation Capture (Hi-C) into the Viral Modulation of Methane Production in Anaerobic Digestion
Source: Environ Sci Technol. 2026 Jul 9;60(28):19866–81. doi: 10.1021/acs.est.6c03952 (PMC13394424; doi:10.1021/acs.est.6c03952)
Supplement: Supplementary file 1 [file es6c03952_si_001.pdf]

Insights gained by high-throughput chromosome conformation capture (Hi-C) into the viral modulation of methane production in anaerobic digestion

Junya Zhang[1,5]*, Tiedong Lu[2]*, Yunwei Cui[1,4], Qihe Tang[1,4], Yuansong Wei[1,5]**, Hans Hermann Richnow[3]**

[1] State Key Laboratory of Regional Environment and Sustainability, Research Center for Eco-Environmental Sciences, Chinese Academy of Sciences, Beijing 100085, China

[2] Agricultural Resource and Environment Research Institute, Guangxi Academy of Agricultural Sciences, Nanning, Guangxi 530007, China

[3] Atmospheric Chemistry Department (ACD), Leibniz Institute of Tropospheric Research (TROPOS), Permoserstraße 15, 04318 Leipzig, Germany

[4] College of Life Science and Technology, Guangxi University, Nanning 530005, China

[5] University of Chinese Academy of Sciences, Beijing 100049, China

* Junya Zhang and Tiedong Lu contributed equally to this work.

****Correspondence:**

Tel.: +86-10-62849690; Fax: +86-10-62843276;

E-mail address: yswei@rcees.ac.cn (Yuansong Wei); richnow@tropos.de (Hans Hermann Richnow).

Summary: 14 pages, 4 texts, 3 graphs.

This file contains 14 pages including Text S1to S4 and Figure S1 to S3.

The Table S1 to S4 are in the Supporting tables (04-Supplementary_tables);

The assembled MAGs, DNA vOTUs, and RNA vOTUs have been deposited into FigShare (https://doi.org/10.6084/m9.figshare.32324328).

Text captions

S1. Identification of RNA vOTUs along with ARGs and host linkage analysis

S2: Auxiliary Metabolic Genes (AMGs) identification

S3. Changes of bacterial community composition and activity in AD

S4. Changs of viral community in AD

Figure captions

Figure S1. The mapped methane metabolism genes from the collected MAGs in the KEGG methane metabolism (map00680).

Figure S2. Changes of microbial community at phylum level (a) and heatmap showing the changes of microbial community at species level (b) along with anaerobic digestion.

Figure S3. Procrustes analysis showing the significant correlation between viral and bacterial community in anaerobic digestion.

Text S1. Identification of RNA vOTUs along with ARGs and host linkage analysis

Two methods were used for the RNA vOTUs identification. One is based on the RNA-directed RNA polymerase (RdRp) homology referred to previous study [1], and the other is based on the gene marker and machine learning through the geNomad [2]. Firstly, RNA viral contigs were identified based on homology searches using profile hidden Markov model approaches (HMMs) of RdRp domains (doi:10.5281/zenodo.5731488). To increase detection of divergent RdRp domain sequences, profile HMMs were generated and updated over ten iterations by recruiting newly detected sequences from our study as described previously [1]. The portion aligning to the HMM was trimmed by using HMMsearch (HMMER 3.1) with the flag -A for the hits with a bit score ≥30. Only sequences longer than 70% of the average length of the best-matching profile HMM were recruited and clustered to generate new HMMs by using the vFam pipeline with default parameters. The original HMM length was kept after each iteration to calculate the length fraction of the footprint. Secondly, the RNA viral contigs was identified through the geNomad with the end-to-end command under the default parameters. Only the viral contigs assigned as the *Riboviria* was remained as the RNA viruses excluding the identified DNA viruses which was expressed revealed by meta-transcriptome. Then, the identified RNA viral contigs by RdRp homology and geNomad were combined and redundancy removed through cd-hit-est (-c 1.0). Finally, the valid 1,435 viral contigs were subjected to species-level clustering to create viral operational taxonomic units (vOTUs) using the ClusterGenomes scripts, following the MIUViG recommended criteria of 90% average nucleotide identity (ANI) and 80%

alignment fraction (AF), resulting in the identification of 517 RNA vOTUs.

The quality of the RNA vOTUs was checked through checkV, and the lifestyle was predicated by VIBRANT. The open reading frames (ORFs) were predicted using Prodigal v2.6.3 (-meta), and then searched against the database of CARD (–query-cover 70, –id 80, –e-value 1e-10) for the identification of ARGs. Taxonomic assignment of these vOTUs was carried out using three methods: 1) vConTACT2 [3]; 2) CAT; and 3) geNomad. We also tried to establish the RNA virus-host linkage based on CRISPR spacer, tRNA, homology match method and RdRp protein sequence similarity to endogenous virus elements (EVEs) between RNA vOTUs and MAGs, although none host linkage between these RNA phages and MAGs was identified.

Text S2: Auxiliary Metabolic Genes (AMGs) identification

The identification and classification of auxiliary metabolic genes (AMGs) in viral genomes remain a subject of considerable debate due to the absence of a standardized definition or identification pipeline. AMGs are typically defined as viral genes, often acquired from host genomes, that augment or redirect host metabolism during infection to enhance host fitness or competitive advantage, thereby indirectly increasing viral fitness. Crucially, AMGs are non-essential for core viral functions such as replication, assembly, or structural integrity. However, determining which genes meet these criteria is challenging, as their roles can vary depending on the specific phage-host interaction and environmental context.

Current annotation tools, such as DRAM-v and VIBRANT, are widely used to identify AMGs in viral sequences, but their databases include genes that some researchers argue do not qualify as AMGs. For instance, genes like *dcm* (K00558, DNA cytosine-5-methyltransferase) and *metK* (K00789, S-adenosylmethionine synthetase) are classified as AMGs by these tools. However, many studies contend that *dcm*, which mediates DNA methylation, primarily serves to protect viral DNA from host restriction-modification (RM) systems, thus fulfilling an essential viral function (e.g., immune evasion) rather than enhancing host metabolism. Similarly, *metK*, involved in S-adenosylmethionine synthesis, supports amino acid metabolism critical for viral protein synthesis, leading some researcher to exclude it from the AMG category. Other debated gene categories include those involved in DNA-related reactions (e.g., nucleotide metabolism), viral invasion (e.g., glycoside hydrolases and peptidases for cell wall lysis), modification of viral components (e.g., glycosyltransferases, adenylyltransferases, and methyltransferases for viral DNA, RNA, or protein modification), structural proteins, ribosomal proteins, transcriptional/translational regulators, and genes unique to eukaryotic systems. These are often considered essential for viral replication or survival and are thus excluded from strict AMG definitions in some studies.

Despite these arguments, we propose that a rigid exclusion of such genes may overlook their potential to enhance host fitness in specific ecological contexts, such as anaerobic digestion (AD). For example, *dcm* may not only protect viral DNA from host defenses

but also stabilize host DNA against environmental stressors, such as thermal stress prevalent in thermophilic AD conditions (e.g., Stage IV, 55°C). In cases where the host lacks an active RM antiviral defense system, *dcm* may not be essential for the virus but could still confer competitive advantages to the host, such as protection against other phages or environmental pressures. Similarly, serine cycle genes like *glyA* (K00600), involved in amino acid metabolism, are debated due to their role in supporting viral replication. However, in methane metabolism studies, genes like *serA* (K00058) and *eno* (K01689) are widely recognized as AMGs because they enhance host metabolic pathways (e.g., serine cycle) that sustain methanogenesis under stress, indirectly benefiting viral propagation through host survival.

The lack of a standardized AMG identification approach complicates these classifications. Strict filtering criteria, which exclude genes associated with nucleotide metabolism, amino acid metabolism, or methylation, risk dismissing genes that perform "legitimate" AMG functions in specific contexts. For instance, if we exclude *glyA* and other serine cycle genes due to their role in amino acid metabolism, we would overlook their documented contributions to methane cycling in AD, as evidenced by their enrichment in lysogenic vOTUs during substrate limitation (Stage III) and thermal stress (Stage IV). A recent perspective emphasizes caution in interpreting viral AMGs, noting that their compact genomes limit coding capacity for accessory genes, making the evolutionary significance of AMGs intriguing. From an evolutionary standpoint, AMGs should prioritize host metabolic augmentation over essential viral needs, but this

distinction is context-dependent.

In this study, we adopted an inclusive approach by relying on DRAM-v and VIBRANT annotations to identify AMGs, acknowledging their limitations but valuing their comprehensive coverage. This decision was driven by the recognition that even debated AMGs, such as *dcm* and *metK*, may contribute to host fitness in the dynamic AD environment, where operational parameters like temperature, total solids (TS), and substrate availability impose selective pressures. For example, *dcm* may enhance host resilience to thermal stress, while *serA* and *eno* support methanogenic pathways critical for methane production. Excluding these genes based solely on their potential "essential" roles in viral replication would neglect their ecological contributions, particularly in stabilizing microbial communities under stress. By retaining DRAM-v and VIBRANT annotations, we ensure a holistic analysis of AMGs' roles in AD, capturing both direct (e.g., methane metabolism via *serA*, *eno*, *fwdH*) and indirect (e.g., stress adaptation *via dcm*) contributions to methane cycling.

Text S3. Changes of bacterial community composition and activity in AD

Bacteroidetes dominated the bacterial community at mesophilic condition, which accounted for 45.2%±11.2%. While Firmicutes became dominant when AD turned into thermophilic condition (74.8%±22.5%), and Thermotogae increased significantly along with the increase of TS (maximum 80.7%) (Figure S2). The key functional species also changed significantly. The dominant functional species at mesophilic condition located on the cluster 1 as *Fermentimonas caenicola*, GGB23723_SGB36506, and etc.. The

dominant methanogens belonged to the *Methanosarcina mazei* and *Methanothrix soehngenii* at mesophilic condition, while they disappeared and the *Methanoculleus thermophilus* and *Methanosarcina thermophilia* became the dominant methanogens at thermophilic condition. Further increase of TS enriched the *Defluviitoga tunisiensis*, *Streptococcus alactolyticus*, *Lactobacillus amylovorus*, etc.. The bacterial diversity was also significantly reduced when AD was turned into the thermophilic condition as indicated by Shannon index, Gini index and Pielou evenness. The PCoA analysis also separate the samples into three clusters based on the changes of temperature and TS, which indicated the significant influence caused by them in AD. We also discovered some species that could be persistent along with the changes of varied operational parameters such as *Corynebacterium pollutisoli*, *Enterococcus SGB6173*, and *Corynebacterium xerosis*, etc.

Because metaphlan4 used the functional biomarker for the taxonomy analysis, not just based on the 16S rDNA, it can reflect the active bacterial community based on the metatranscriptome, although the rRNA has been removed during the preparation for the sequencing. The dominant phylum also belonged to the Bacteroidetes (38.4%) at mesophilic condition at RNA level, and the active phylum belonged to Firmicutes (76.5%) when AD was turned into thermophilic condition, while Thermotogae (58.3%) became dominant when the TS was increased. The active microbial community showed similar pattern with that at the DNA level. PCoA analysis of the active bacterial community also indicated the significant impact caused by temperature and TS, although the diversity of active bacterial community showed no significant difference

along with varied operational parameters even between mesophilic and thermophilic condition ($p>0.05$). The key functional species also showed similar pattern, where the dominant active bacterial community belonged to GGB23094_SGB35645 (23.9%), GGB42812_SGB58388 (13.2%), etc. The active methanogens belonged to *Methanothrix soehngenii* (10.4%), *Methanocullecus*_SGB45879 (1.9%) and *Methanosarcina_mazei* at mesophilic condition, while it was *Methanocullues_thermophilus* (4.4%) at thermophilic condition. The species belonging to *Corynebacterium xerosis*, *Corynebacterium pollutisoli* and *Escherichia coli* showed activity no matter of the condition in AD. *Defluviitoga_tunisiensis* (58.3%) only showed higher activity at thermophilic condition.

Text S4. Changs of viral community in AD

We identified 6,210 vOTUs in this study, and 17 vOTUs was considered to infect Eukaryota showing the pathogenic potential to human, for example vOTU204 (classified as *Ackermannviridae*), vOTU27 (classified as *Mimiviridae*), and others. The number of observed vOTUs reached saturation with the current sample size and sequencing depth, which indicated that the identified vOTUs here could well reflect the viral community in AD of swine manure. 69.5%±6.6% of the vOTUs belonged to lytic. We further identified 96,069 viral ORFs (vORFs) in this study, which showed some novelty, where only 23.7% of vORFs could find their homology in NCBI virus refseq, while 74.5% of them could find the homology in IMG/VR 3.0. Most of vOTUs (69.5± 6.6%) belonged to the lytic phages, and varied operational parameters showed limited impact on the lifestyle of phages.

Although five methods were used for the taxonomy analysis, 90% of the vOTU could not be assigned into a family. Most of the virus (>95%) belonged to the *Caudoviricetes*, and *Herelleviridae* (8.6%), *Suoliviridae* (7.0%) along with *Straboviridae* (4.0%) were the dominant at Stage I. The additives did not change the viral community significantly, while *Zierdtviridae* was enriched from 0.3% to 1.35%. So did the SRT, but *Schitoviridae* was increased from 0.8% to 2.14%. The thermophilic condition also reshaped the viral community, where the *Straboviridae*, *Steigvirdae* and *Matshushitaviridae* became the dominant. The increase of TS to 15% contribute little to the viral community, but the TS of 20% make the dominance of *Autographiviridae*. The known vOTUs at family level could only reflect part of the variance caused by changes of operational parameters. PCoA analysis based on the vOTUs indicated significant difference caused by temperature and TS in AD, which showed similar patter with bacterial community. Only 17.6% of the vOTUs was shared in AD, and the vOTUs only detected in the thermophilic condition accounted for 51.7%.

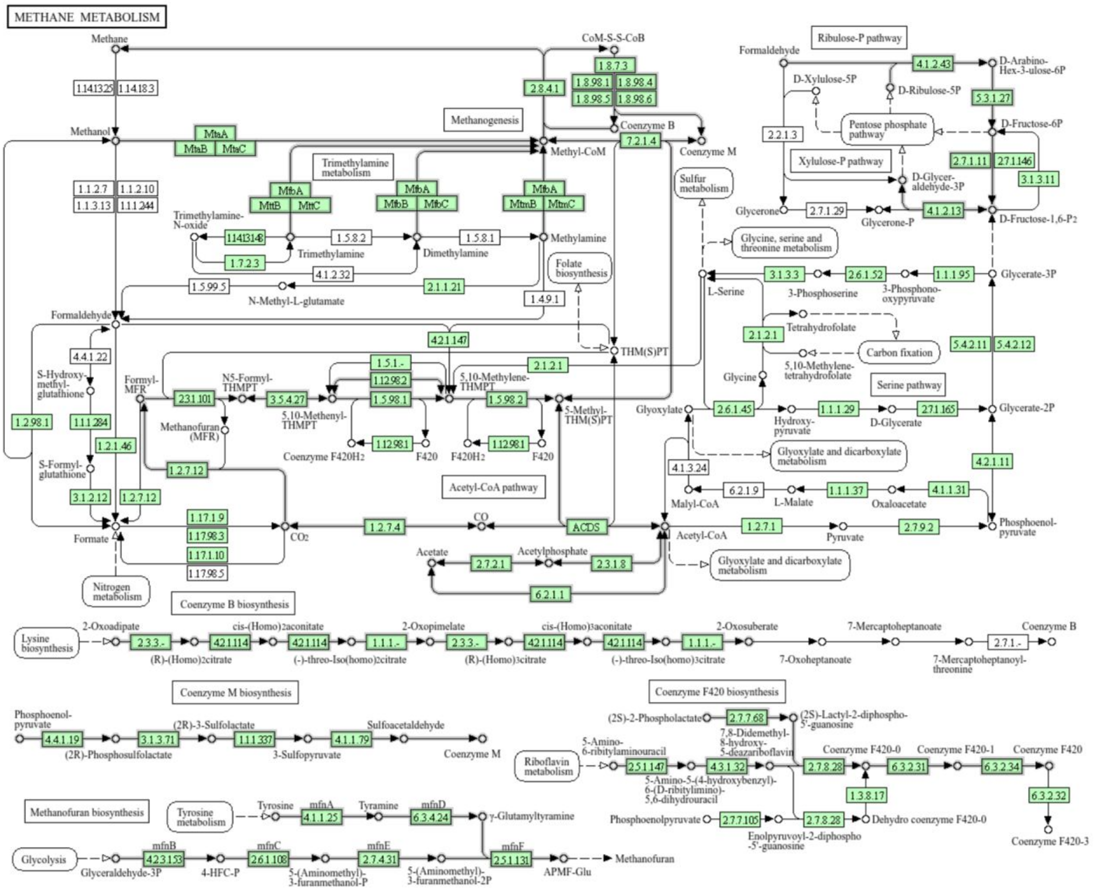

Figure S1. The mapped methane metabolism genes from the collected MAGs in the KEGG methane metabolism (map00680). The green indicated the methane metabolism genes responsible for the enzyme were detected from the collected MAGs in this study.

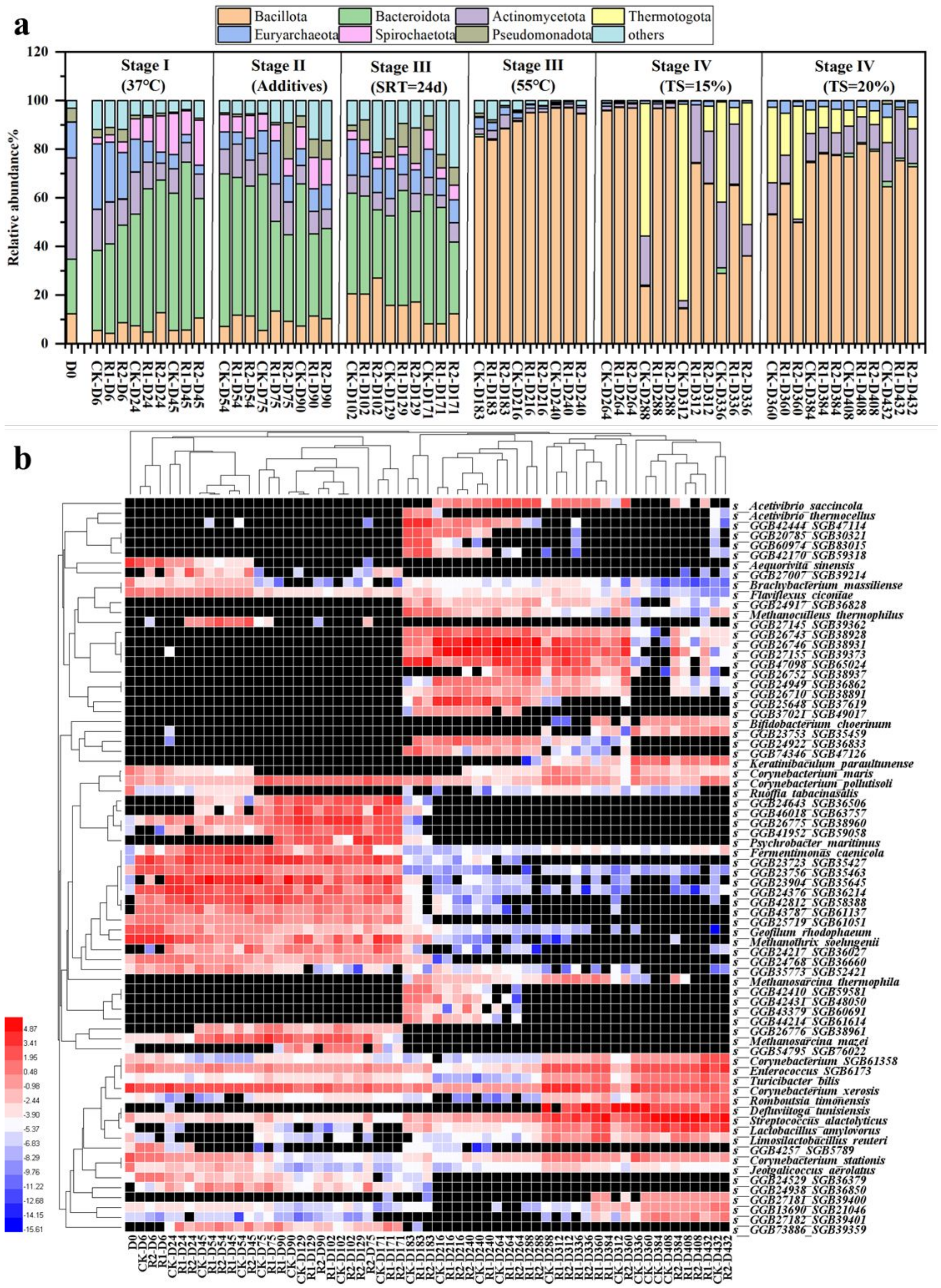

Figure S2. Changes of microbial community at phylum level (a) and heatmap showing the changes of microbial community at species level (b) along with anaerobic digestion.

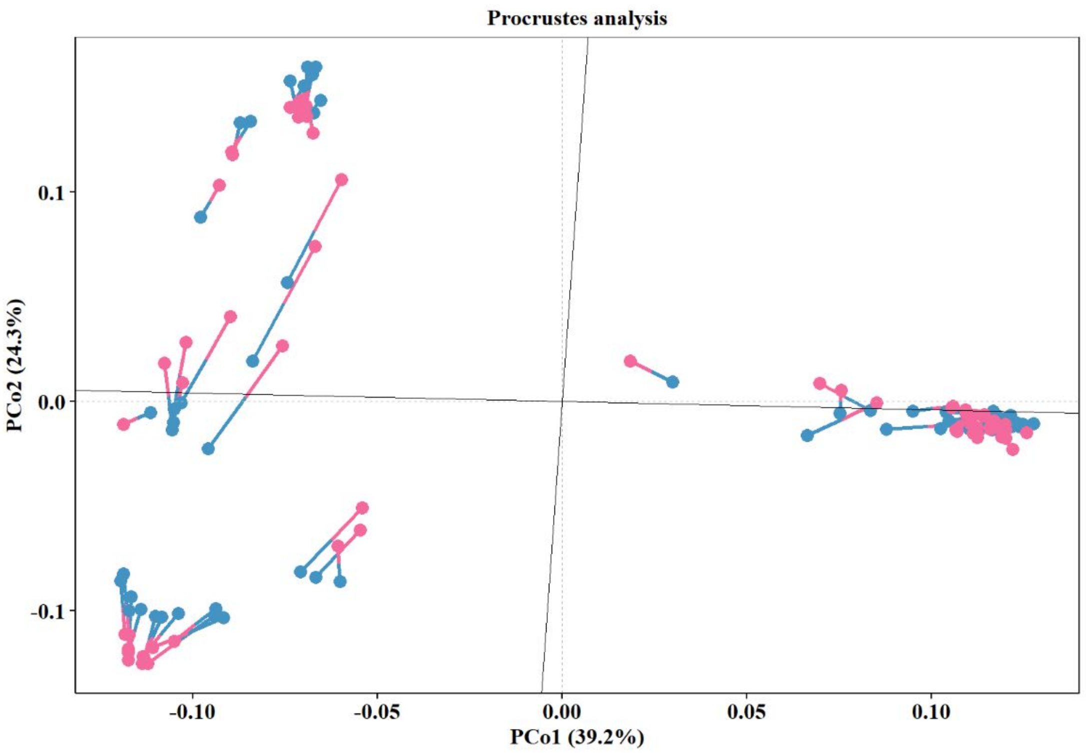

Figure S3. Procrustes analysis showing the significant correlation between viral and bacterial community in anaerobic digestion (Mantel test, rho = 0.986, P < 0.0001).

References

(1) Zayed, A. A.; Wainaina, J. M.; Dominguez-Huerta, G.; Pelletier, E.; Guo, J.; Mohssen, M.; Tian, F.; Pratama, A. A. A.; Bolduc, B.; Zablocki, O.; Cronin, D.; Solden, L.; Delage, E.; Alberti, A.; Aury, J.-M. M.; Carradec, Q.; da Silva, C.; Labadie, K.; Poulain, J.; Ruscheweyh, H. J.; Salazar, G.; Shatoff, E.; Tara Oceans, C.; Bundschuh, R.; Fredrick, K.; Kubatko, L. S.; Chaffron, S.; Culley, A. I.; Sunagawa, S.; Kuhn, J. H.; Wincker, P.; Sullivan, M. B.; Coordinators, T. O. Cryptic and Abundant Marine Viruses at the Evolutionary Origins of Earth's RNA Virome. *Science.* **2022**, *376* (6589), 156–162. https://doi.org/10.1126/science.abm5847.

(2) Camargo, A. P.; Roux, S.; Schulz, F.; Babinski, M.; Xu, Y.; Hu, B.; Chain, P. S. G.; Nayfach, S.; Kyrpides, N. C. Identification of Mobile Genetic Elements with GeNomad. *Nat. Biotechnol.* **2023**, *42*, 1303–1312. https://doi.org/10.1038/s41587-023-01953-y.

(3) Bin Jang, H.; Bolduc, B.; Zablocki, O.; Kuhn, J. H.; Roux, S.; Adriaenssens, E. M.; Brister, J. R.; Kropinski, A. M.; Krupovic, M.; Lavigne, R.; Turner, D.; Sullivan, M. B. Taxonomic Assignment of Uncultivated Prokaryotic Virus Genomes Is Enabled by Gene-Sharing Networks. *Nat. Biotechnol.* **2019**, *37* (6), 632–639. https://doi.org/10.1038/s41587-019-0100-8.
